# Supplementary material for: USP52 acts as a deubiquitinase and promotes histone chaperone ASF1A stabilization
Source: Nat Commun. 2018 Mar 29;9:1285. doi: 10.1038/s41467-018-03588-z (PMC5876348; doi:10.1038/s41467-018-03588-z)
Supplement: Supplementary file 1 — Supplementary Information(PDF 12924 kb) [file 41467_2018_3588_MOESM1_ESM.pdf]

Supplementary Information

USP52 Acts as a Deubiquitinase and Promotes Histone Chaperone ASF1A Stabilization

Yang et al.

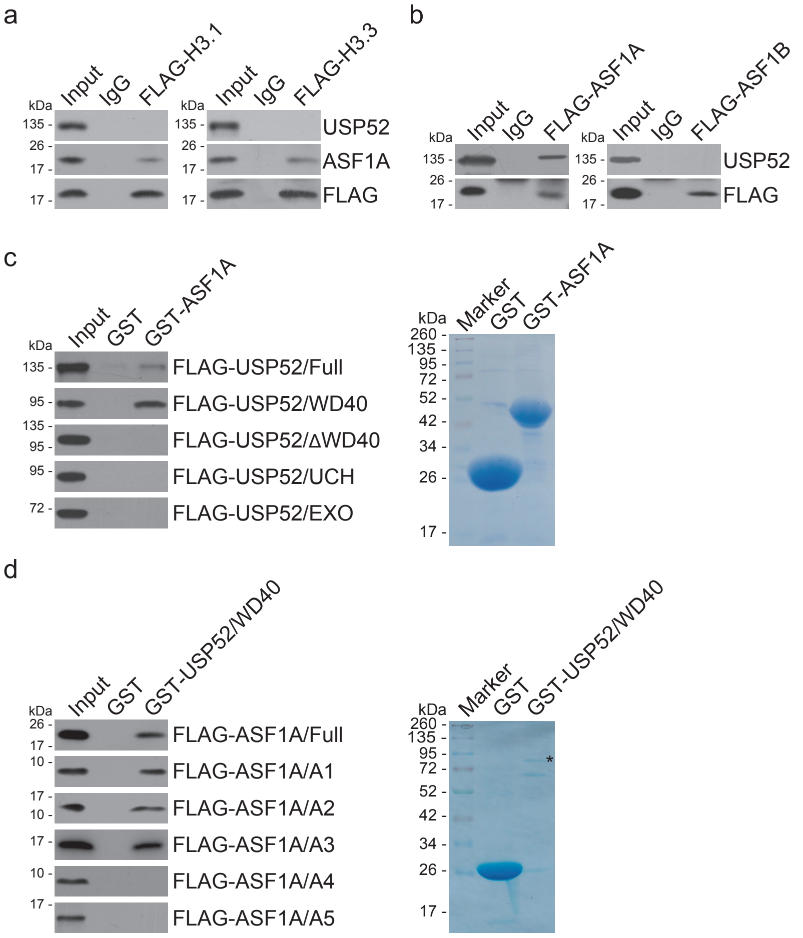

Supplementary Figure 1 (Figure 1 continued). Histone Chaperone ASF1A Is Physically Associated with USP52. (a) Co-immunoprecipitation analysis of the interaction of USP52 or ASF1A with histone H3 using cellular lysates from HeLa cells expressing FLAG-HA-H3.1 or FLAG-HA-H3.3. (b) Co-immunoprecipitation analysis of the interaction of USP52 with the indicated proteins with cellular lysates from HeLa cells expressing FLAG-ASF1A or FLAG-ASF1B. (c) Pull-down analysis of the domains involved in the interaction between ASF1A and USP52 with *in vitro* transcribed/translated deletion mutants of FLAG-GFP tagged USP52 and GST tagged full length ASF1A purified from bacteria cells. (d) Pull-down analysis of the domains involved in the interaction between ASF1A and USP52 with *in vitro* transcribed/translated deletion mutants of FLAG tagged ASF1A and GST tagged WD40 repeat domain of USP52 (USP52/WD40) purified from bacteria cells. The asterisk indicates the recombinant protein stained by Commassie Blue.

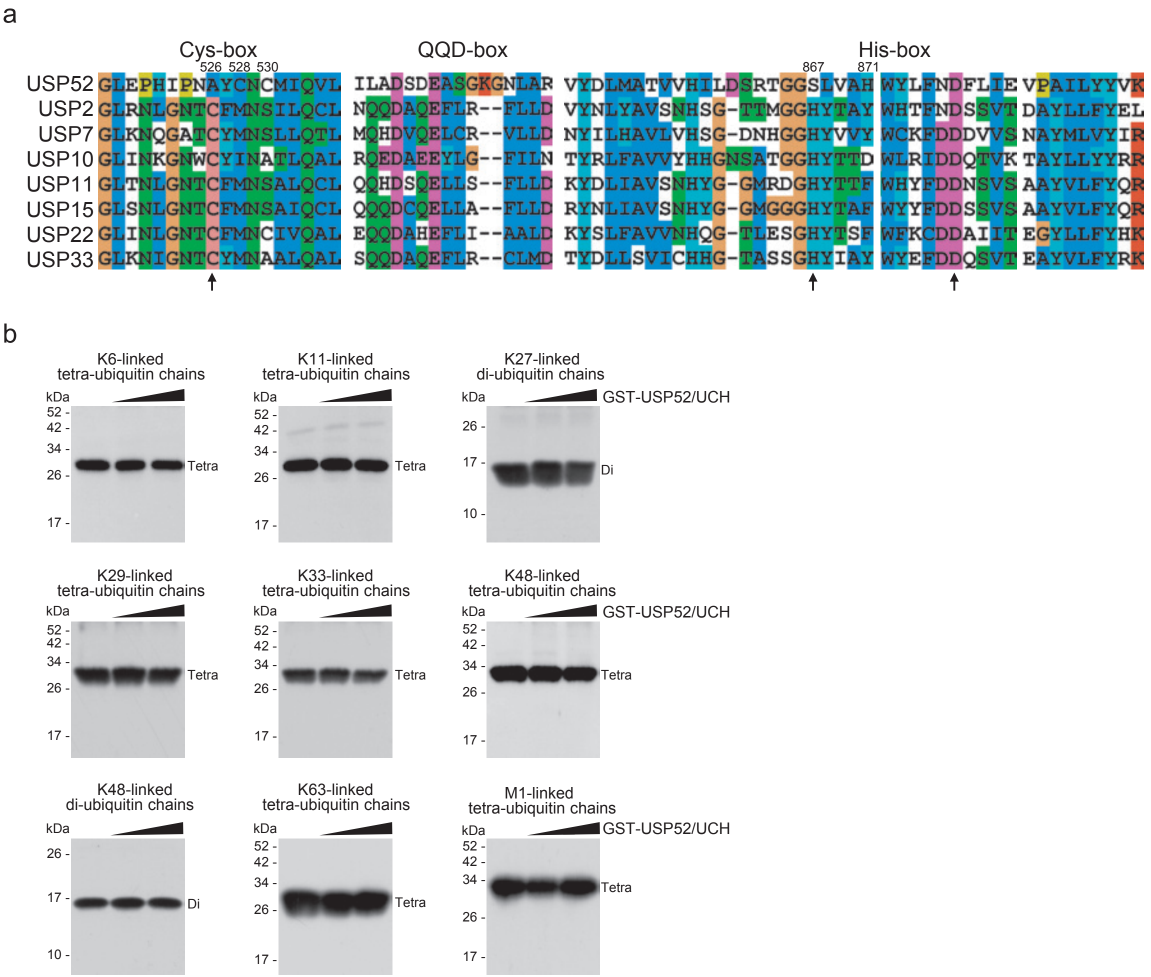

Supplementary Figure 2 (Figure 2 continued). Bacterially Expressed USP52 Has No Deubiquitinase Activity. (a) Sequence alignment of USP52 with other well-characterized DUBs (*Homo sapiens*). The multiple alignments were performed with the CLUSTALX program. Gaps are indicated by hyphens and common residues to all sequences are shadowed. The Cys, His, and Asp/Asn residues characteristic of these cysteine proteinases are indicated with an arrow. (b) *In vitro* deubiquitination assays with increasing amounts of *E.coli* cells-purified UCH domain of USP52 (USP52/UCH, 1 μg and 3 μg) and different types ubiquitin linkages (1 μg). After 4 hours of incubation, the cleavage effect was examined by Western blotting with antibody against ubiquitin.

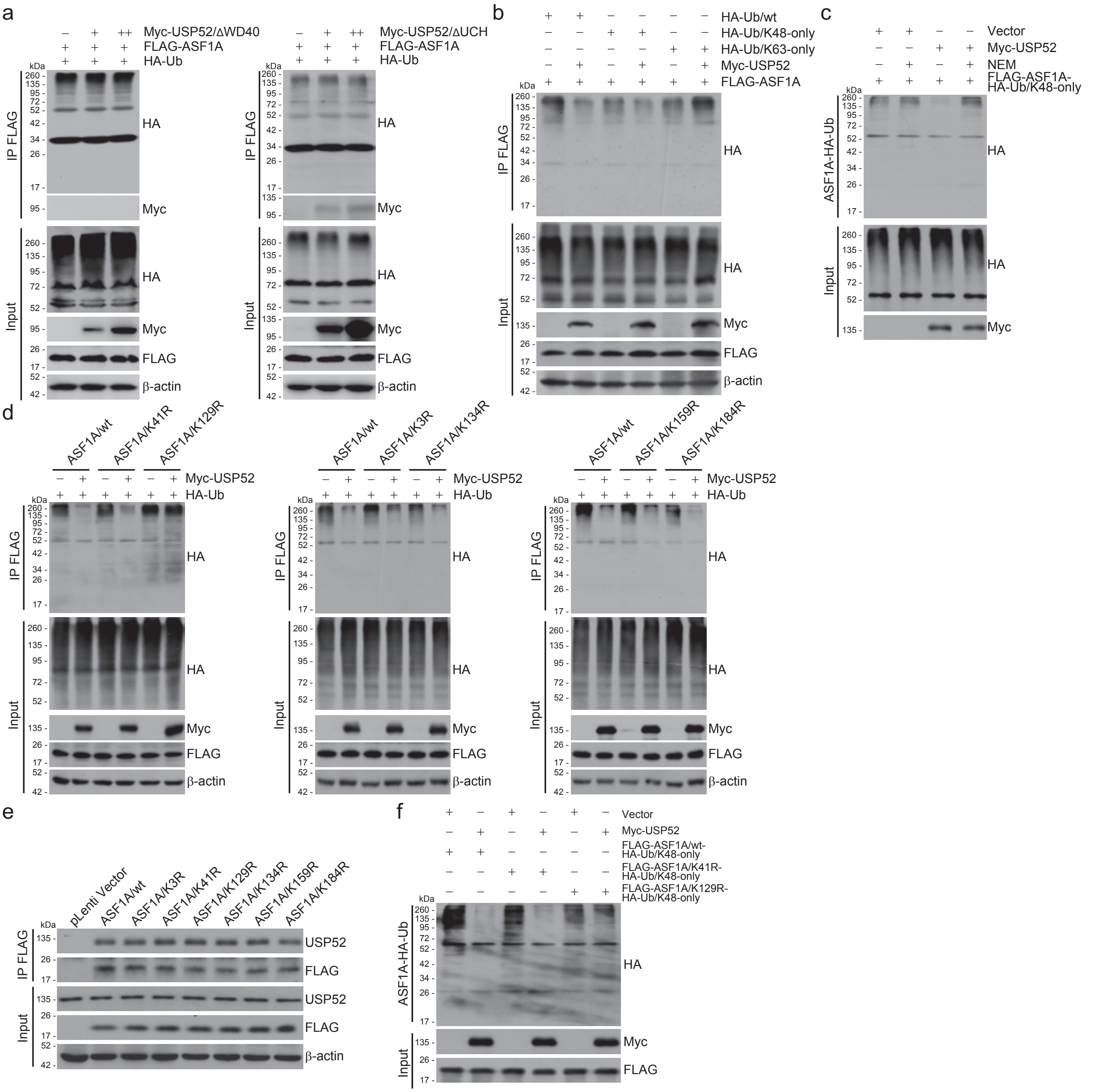

Supplementary Figure 3 (Figure 3 continued). USP52 Deubiquitinates ASF1A. (a) HeLa cells stably expressing FLAG-ASF1A were co-transfected with HA-Ub and different amounts of Myc-USP52/ΔWD40 or Myc-USP52/ΔUCH. Cellular extracts were prepared for co-immunoprecipitation assays with anti-FLAG followed by IB with anti-HA. (b) HeLa cells stably expressing FLAG-ASF1A were co-transfected with Myc-USP52 and HA-Ub, HA-Ub/K48-only or HA-Ub/K63-only as indicated. Cellular extracts were prepared for co-immunoprecipitation assays with anti-FLAG followed by IB with anti-HA. (c) *In vitro* deubiquitination assays in the absence or presence of NEM (2 mM). FLAG-tagged ASF1A-Ub/K48-only and Myc-USP52 (2 μg) were purified from Hela cells with high salt and detergent buffer. (d) HeLa cells stably expressing wild type ASF1A (ASF1A/wt) or different K to R mutants were transfected with control vector or Myc-USP52 as indicated. Cellular extracts were prepared for co-immunoprecipitation assays with anti-FLAG followed by IB with anti-HA. (e) HeLa cells stably expressing FLAG tagged ASF1A/wt or different K to R mutants were collected for co-immunoprecipitation analysis with antibodies against the indicated proteins. (f) *In vitro* deubiquitination assays with HeLa cells-purified Myc-USP52 (2 μg) and HA-Ub/K48-only conjugated FLAG-tagged ASF1A/wt, ASF1A/K41R or ASF1A/K129R by high salt and detergent buffer.

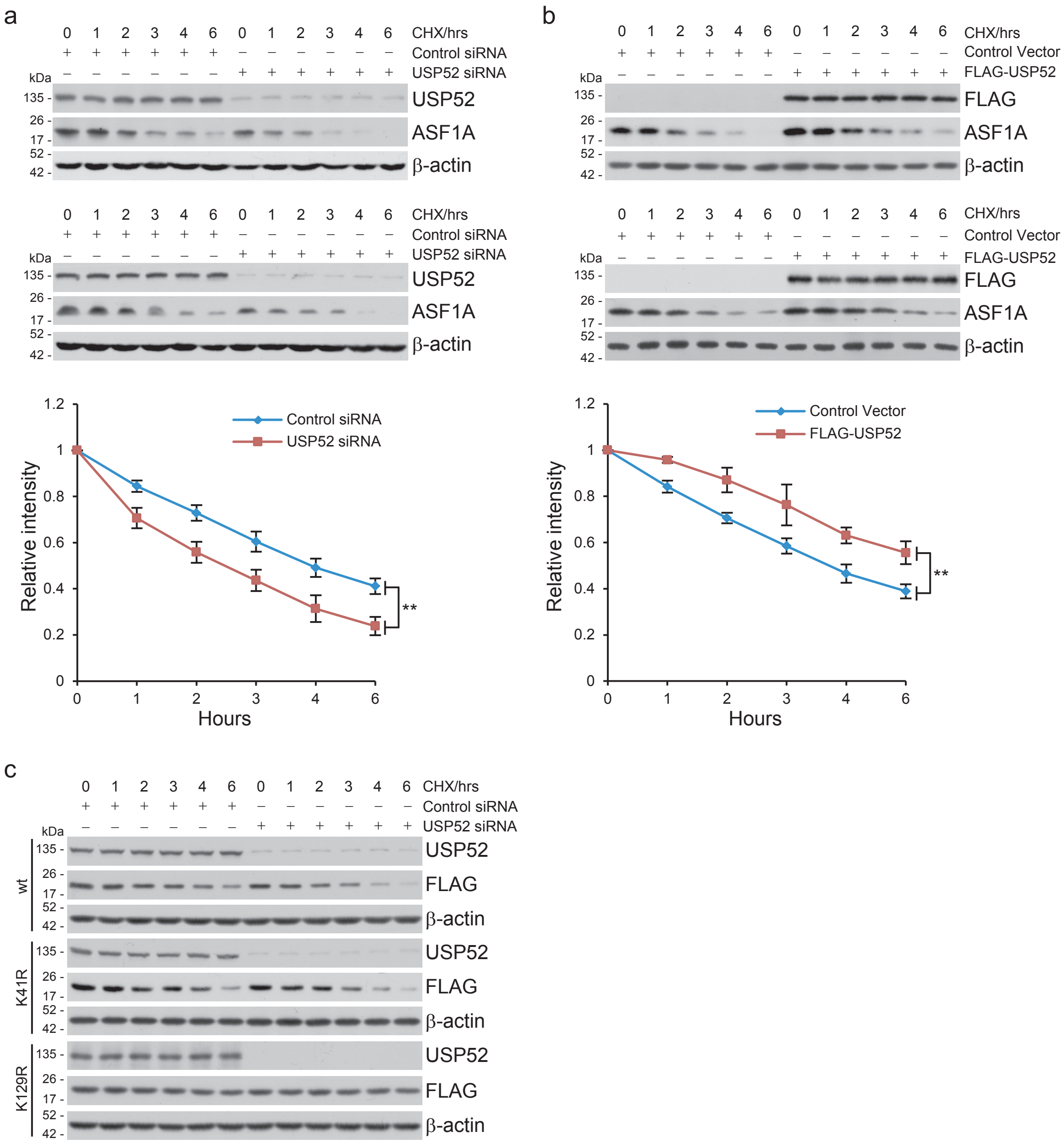

Supplementary Figure 4 (Figure 4 continued). USP52 Promotes ASF1A Stabilization. (a) MCF-7 cells transfected with control siRNA or USP52 siRNA were treated with cycloheximide (CHX) and harvested at the indicated time followed by Western blotting analysis. Two sets of representative images from biological triplicate experiments are shown (Related to the upper panel of Fig. 4g). The intensity of each band from biological triplicate experiments was quantified by densitometry with Image J software with β-actin as a normalizer. Each bar represents the mean ± S.D. for biological triplicate experiments. $^{**}P<0.01$, two-way ANOVA. (b) MCF-7 cells stably expressing control vector or FLAG-USP52 were treated with cycloheximide (CHX) and harvested at the indicated time followed by Western blotting analysis. Two sets of representative images from biological triplicate experiments are shown (Related to the lower panel of Fig. 4g). The intensity of each band from biological triplicate experiments was quantified by densitometry with Image J software with β-actin as a normalizer. Each bar represents the mean ± S.D. for biological triplicate experiments. $^{**}P<0.01$, two-way ANOVA. (c) MCF-7 cells stably expressing ASF1A/wt, ASF1A/K41R or ASF1A/K129R mutant were transfected with control siRNA or USP52 siRNA. Cells were treated with CHX and harvested at the indicated time followed by Western blotting analysis.

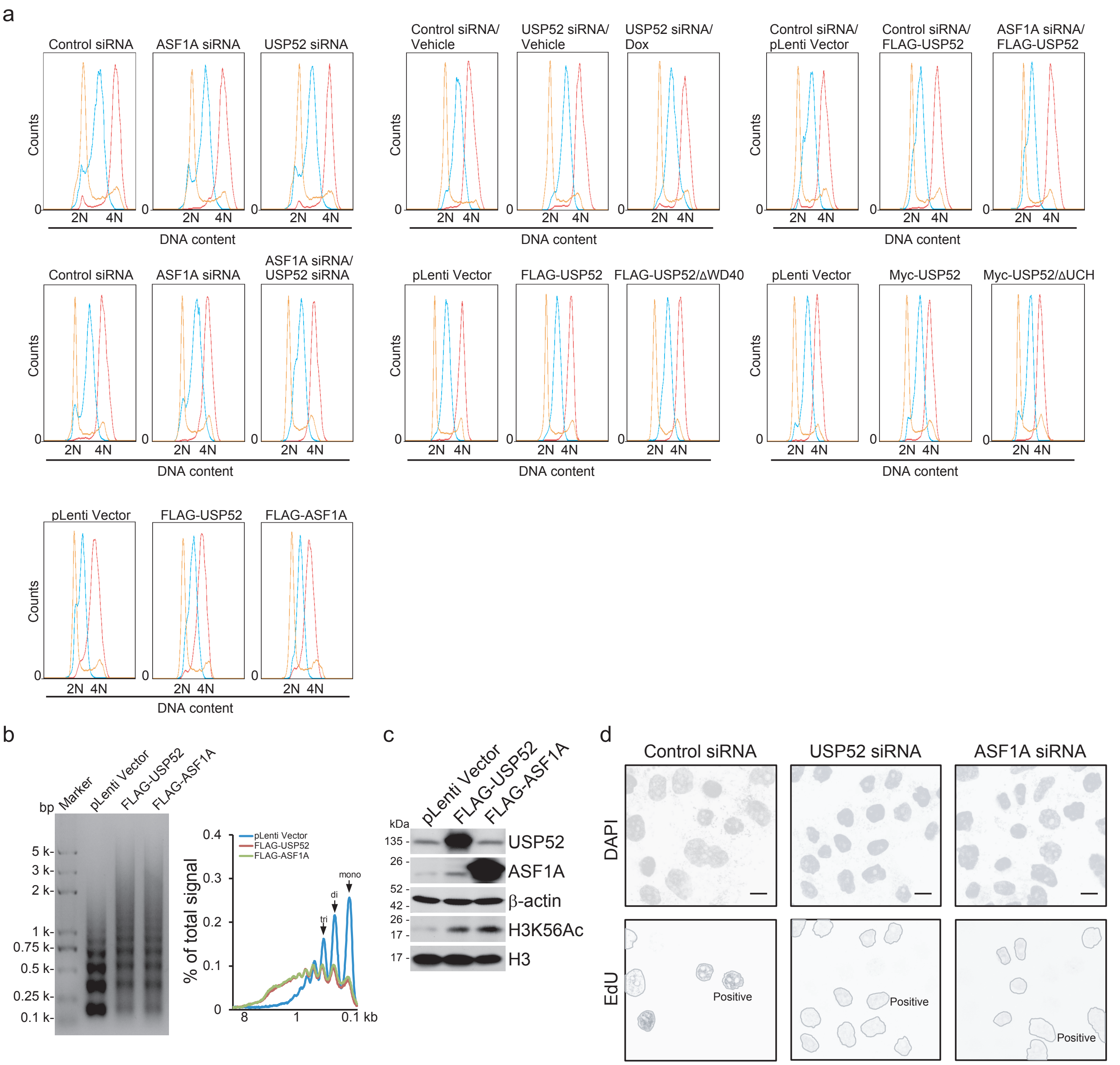

Supplementary Figure 5 (Figure 5 continued). USP52 Promotes Chromatin Assembly through Stabilizing ASF1A. (a) Control HeLa cells or HeLa cells stably expressing the indicated genes were transfected with the indicated siRNAs and synchronized by nocodazole followed by drug removal and FACS analysis at 2 hours interval. Cell cycle profiles after synchronization and release in S or M phase are shown. In panel 2, cells with Dox-inducible expression of ASF1A were transfected with control siRNA or USP52 siRNA in the presence or absence of doxycycline. (b) MNase digestion assay. Control HeLa cells or HeLa cells stably expressing FLAG-USP52 or FLAG-ASF1A were synchronized by nocodazole followed by drug removal and FACS analysis at 2 hours interval. The same amounts of isolated nuclei from S phase cells were treated with MNase (0.4 gel Unit/μl) and purified DNAs were resolved in 1.5% agarose gels followed by EtBr staining. MNase digestion patterns were quantified by densitometry and the positions of mono-, di-, or, tri-nucleosomal DNA fragment are indicated. (c) Cellular lysates from cells in (b) were analyzed by Western blotting with antibodies against the indicated proteins. (d) Immunofluorescence analysis of HeLa cells transfected with control siRNA, USP52 siRNA or ASF1A siRNA followed by EdU pulse labelling (related to Fig. 5c). The images were converted to black and white and EdU positive cells were circled as indicated.

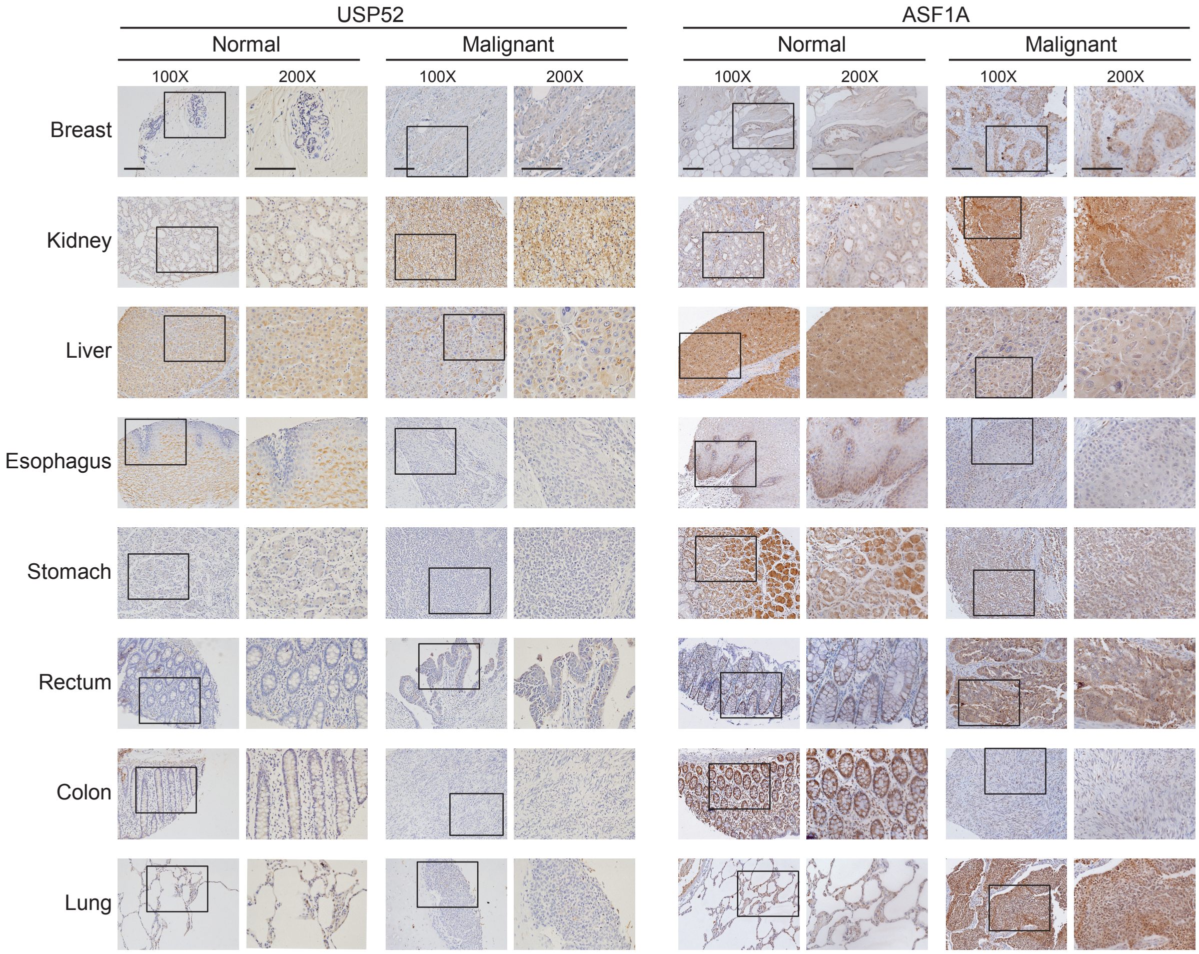

Supplementary Figure 6 (Figure 6 continued). IHC Analysis of the Expression of USP52 and ASF1A in Multiple Tumor Samples. Human tissue arrays including series of tumor samples from breast, kidney, liver, esophagus, stomach, rectum, colon, and lung with each type of cancer having three malignant samples paired with adjacent normal tissues, were used to examine the expression profiles of USP52 and ASF1A. Representative images (100 X and 200 X magnification) of immunohistochemical (IHC) stainings are shown. Scale bar, 50 μm.

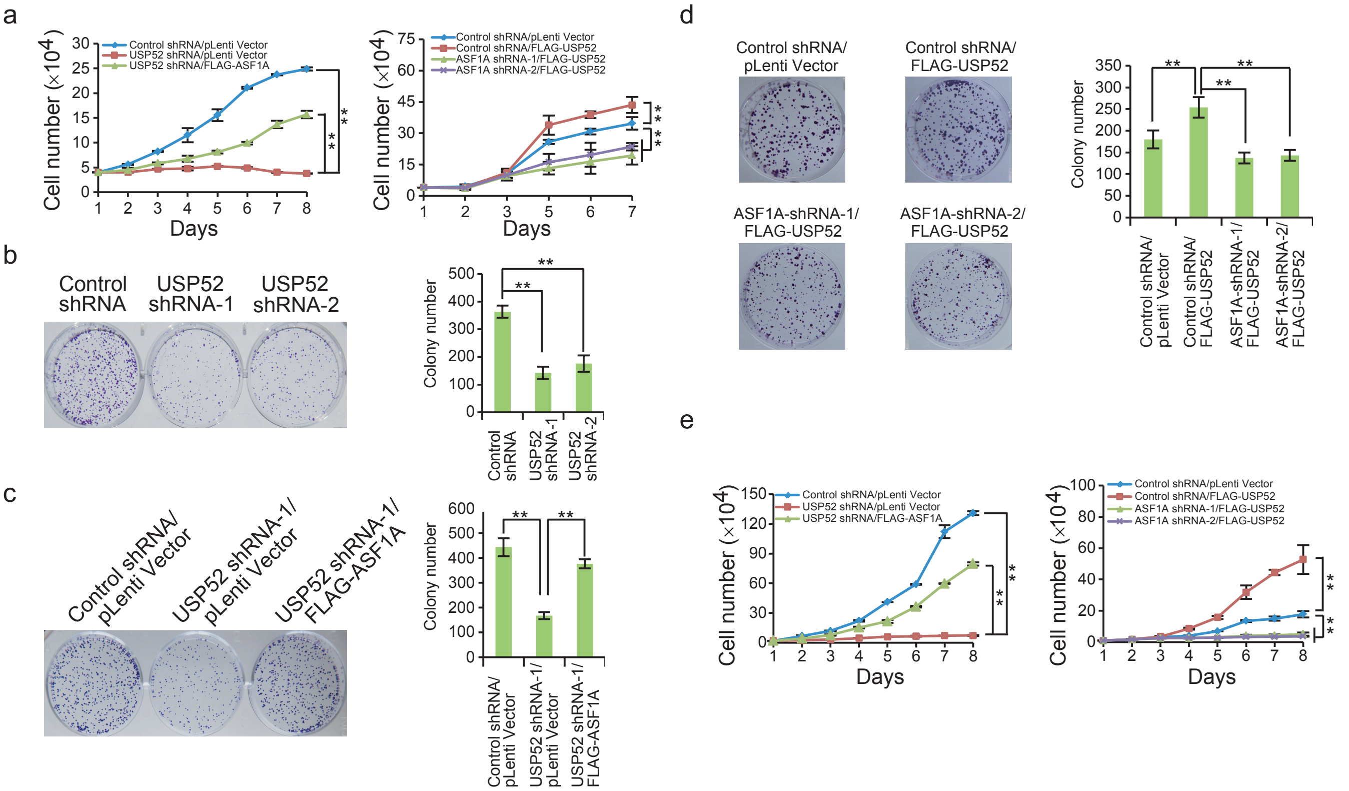

Supplementary Figure 7 (Figure 6 continued). USP52/ASF1A Signaling Axis Is Required for Breast Cancer Cell Proliferation. (a) MCF-7 cells stably expressing the indicated genes or shRNAs were subjected to growth viability assay. Each bar represents the mean ± S.D. for biological triplicate experiments. $^{**}P<0.01$, two-way ANOVA. (b) Colony formation assays of ZR-75-1 cells stably expressing USP52 shRNAs. Representative images from biological triplicate experiments are shown. $^{**}P<0.01$, one-way ANOVA. (c) Colony formation assays of ZR-75-1 cells stably expressing the indicated genes and shRNAs. Representative images from biological triplicate experiments are shown. $^{**}P<0.01$, one-way ANOVA. (d) Colony formation assays of ZR-75-1 cells stably expressing the indicated genes and shRNAs. Representative images from biological triplicate experiments are shown. $^{**}P<0.01$, one-way ANOVA. (e) ZR-75-1 cells stably expressing the indicated genes and shRNAs were subjected to growth viability assay. Each bar represents the mean ± S.D. for biological triplicate experiments. $^{**}P<0.01$, two-way ANOVA.

Uncropped blots related to Figure 1b

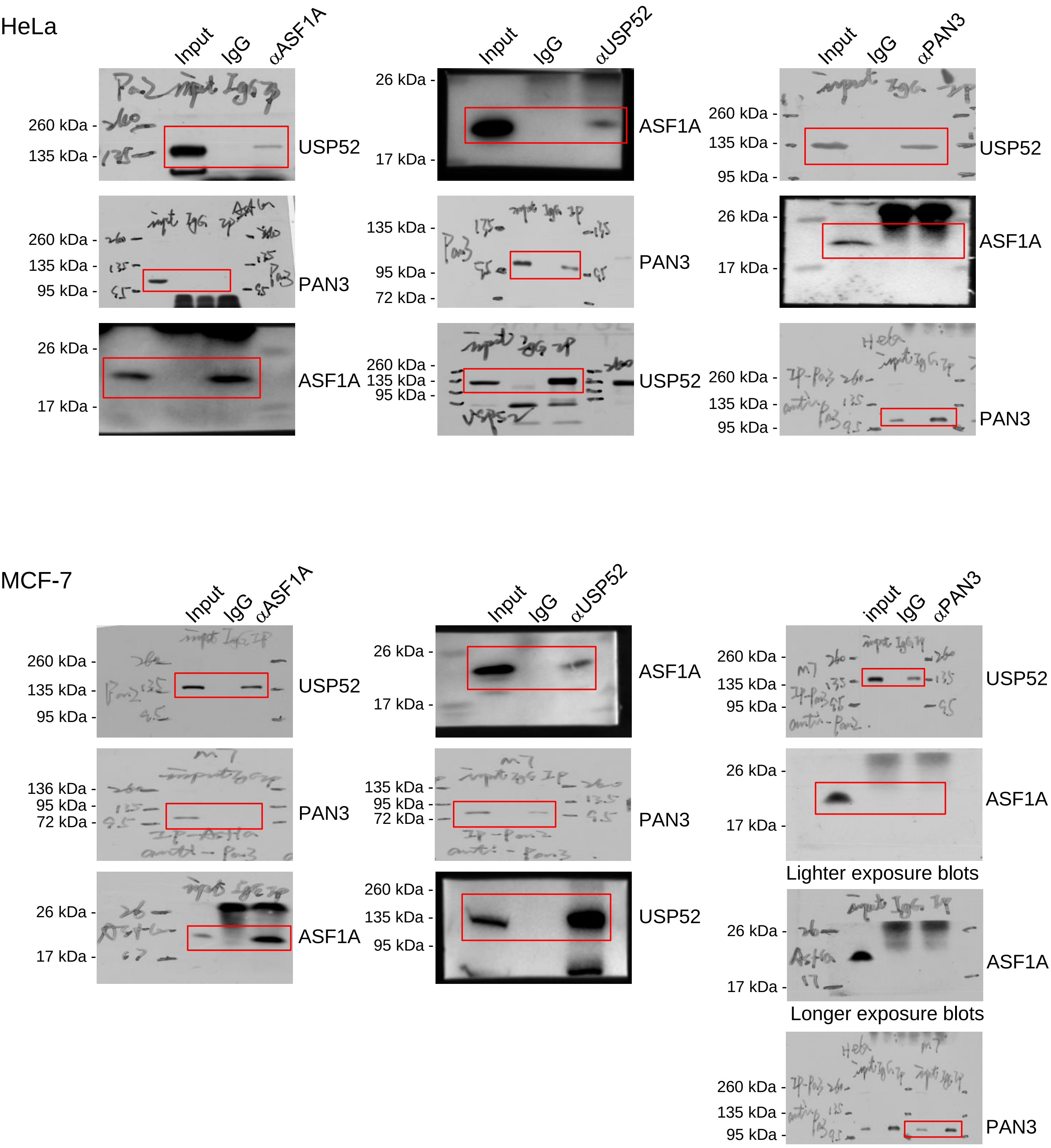

Uncropped blots related to Figure 1c

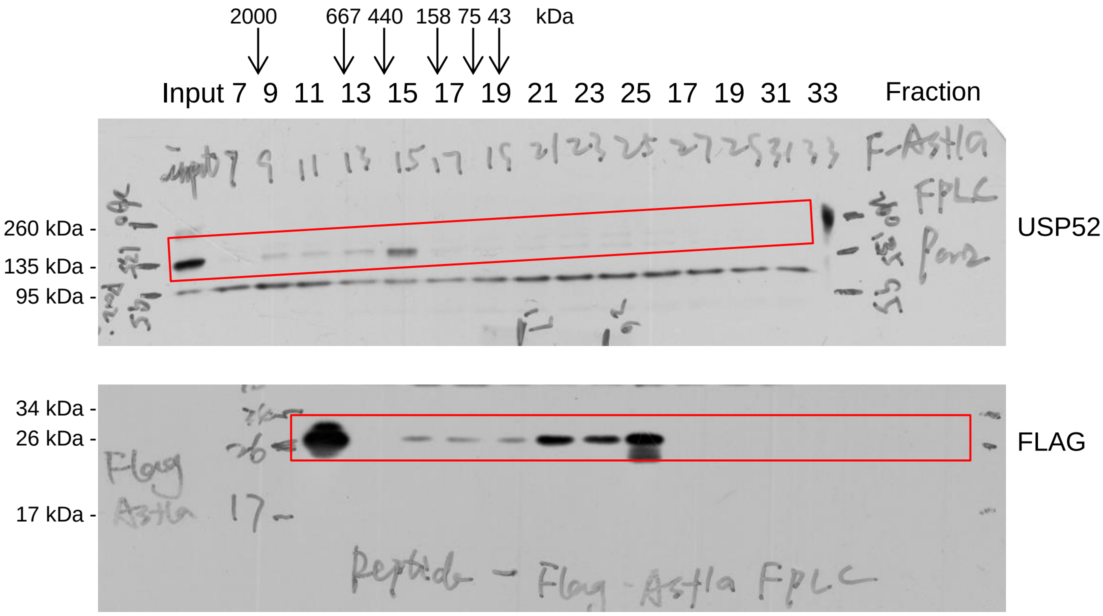

Uncropped blots related to Figure 1d

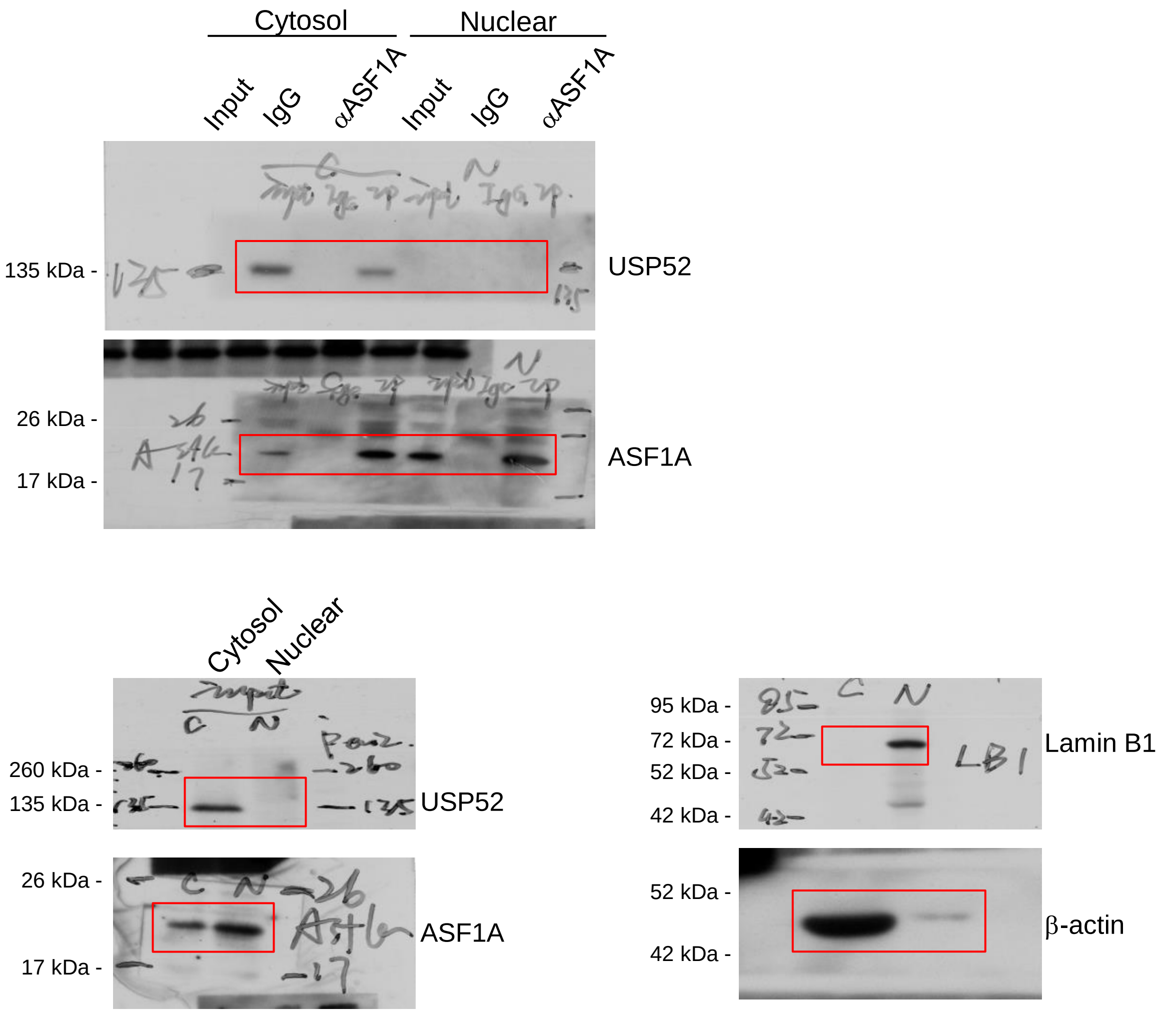

Uncropped blots related to Figure 1f

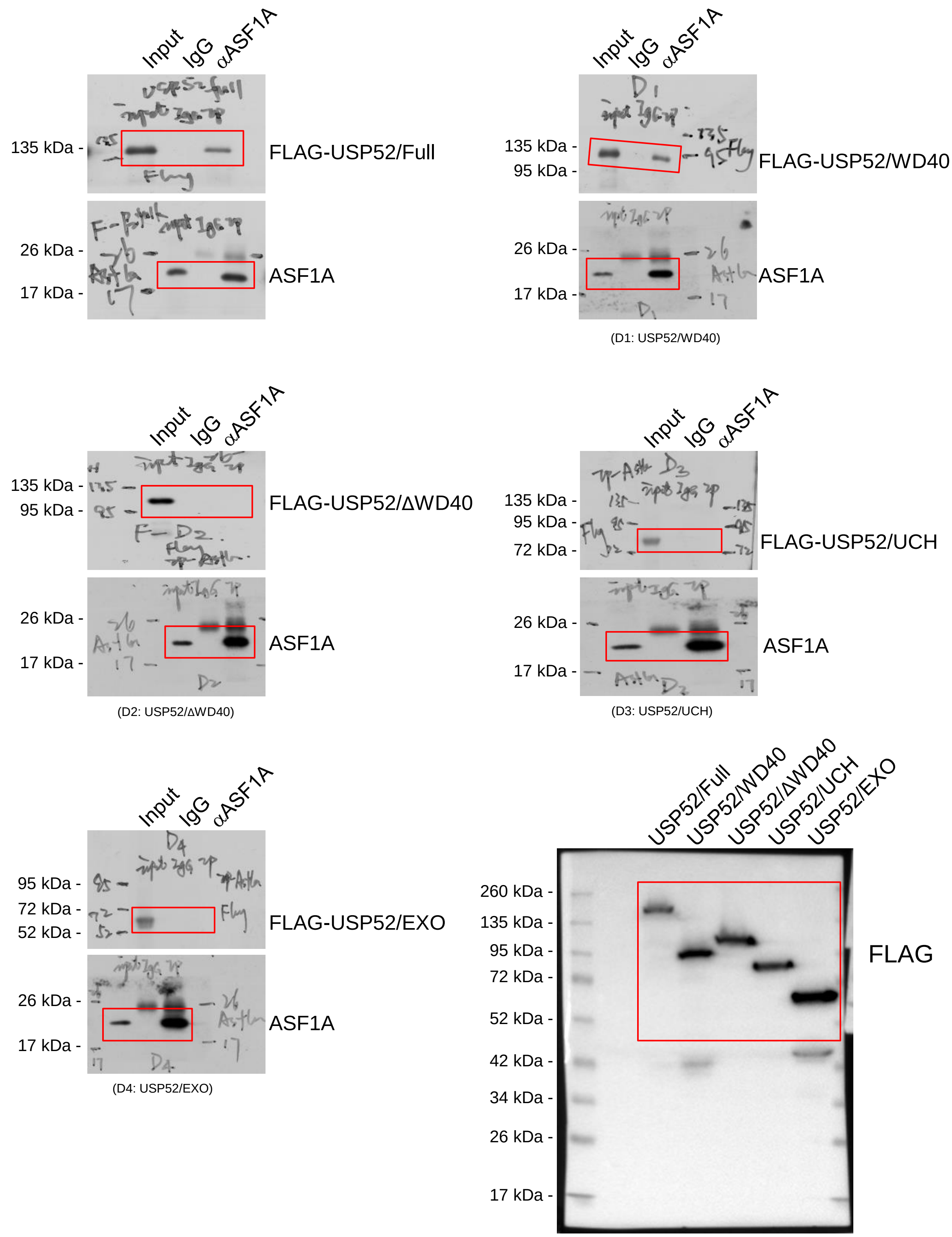

Uncropped blots related to Figure 1g

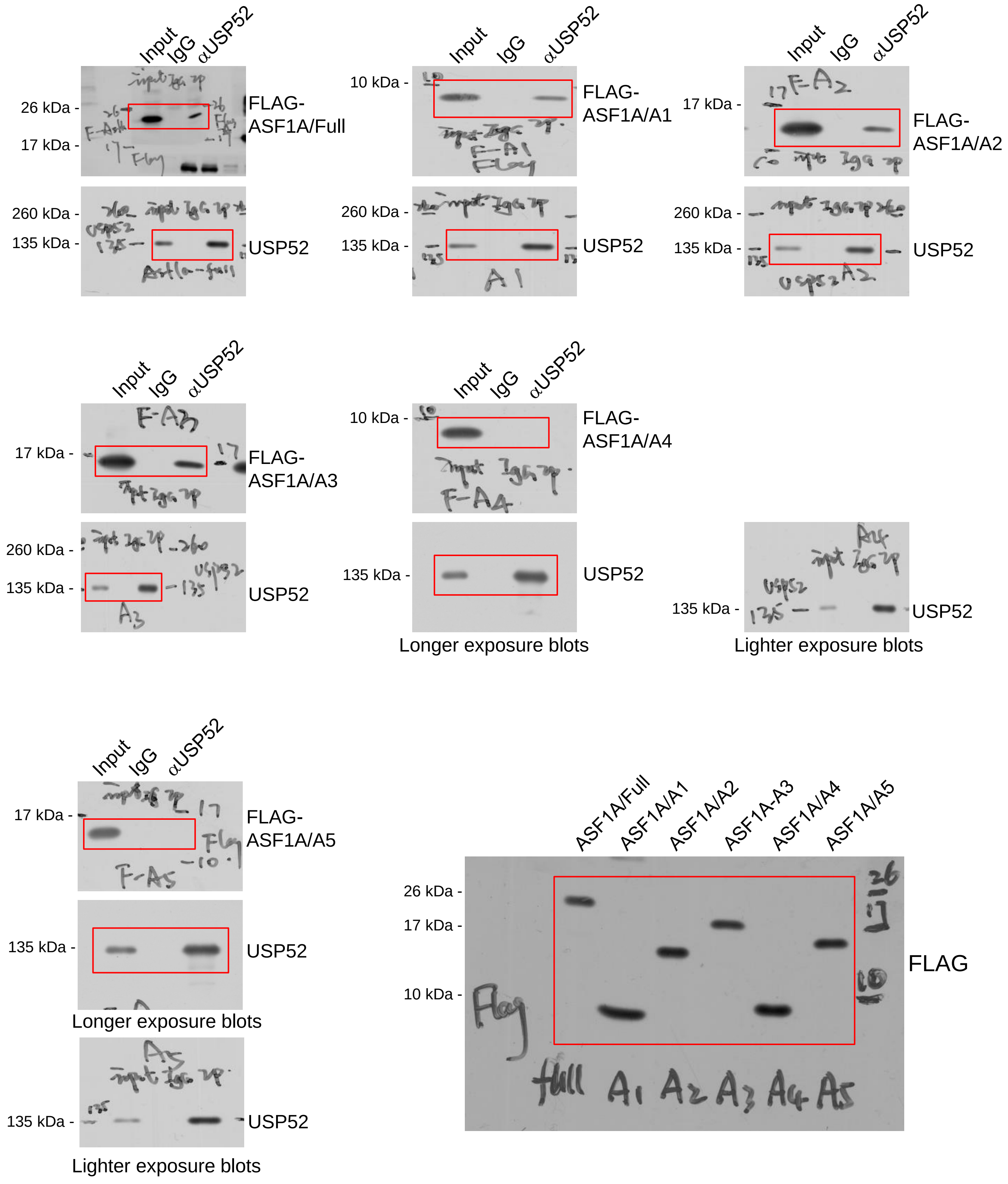

Uncropped blots related to Figure 2a

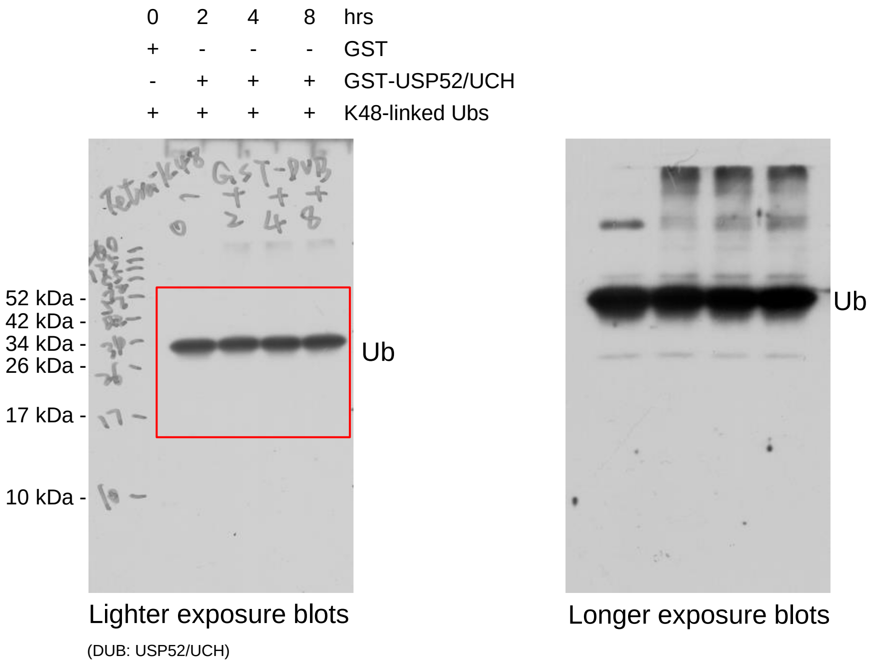

Uncropped blots related to Figure 2b

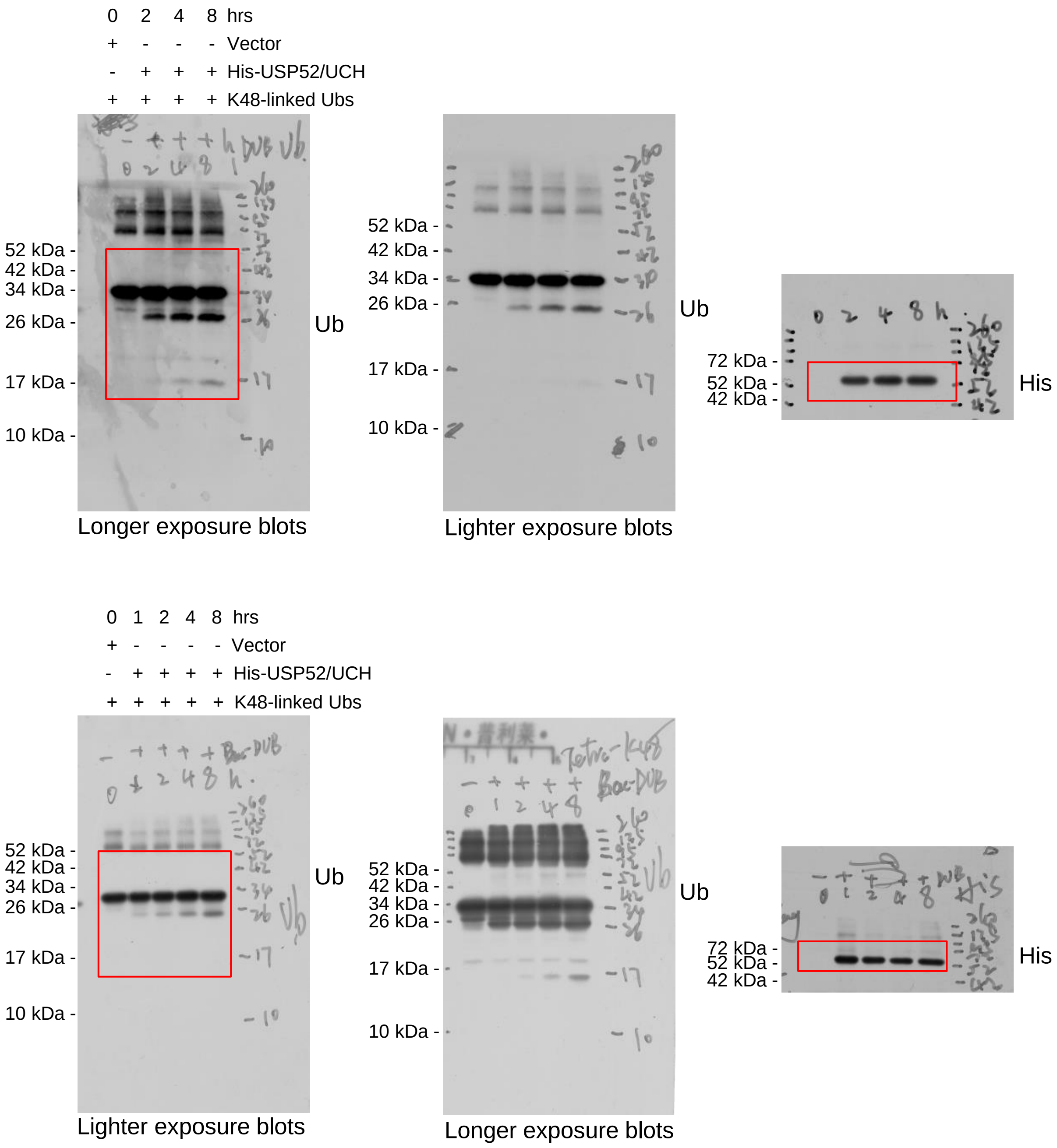

Uncropped blots related to Figure 2c

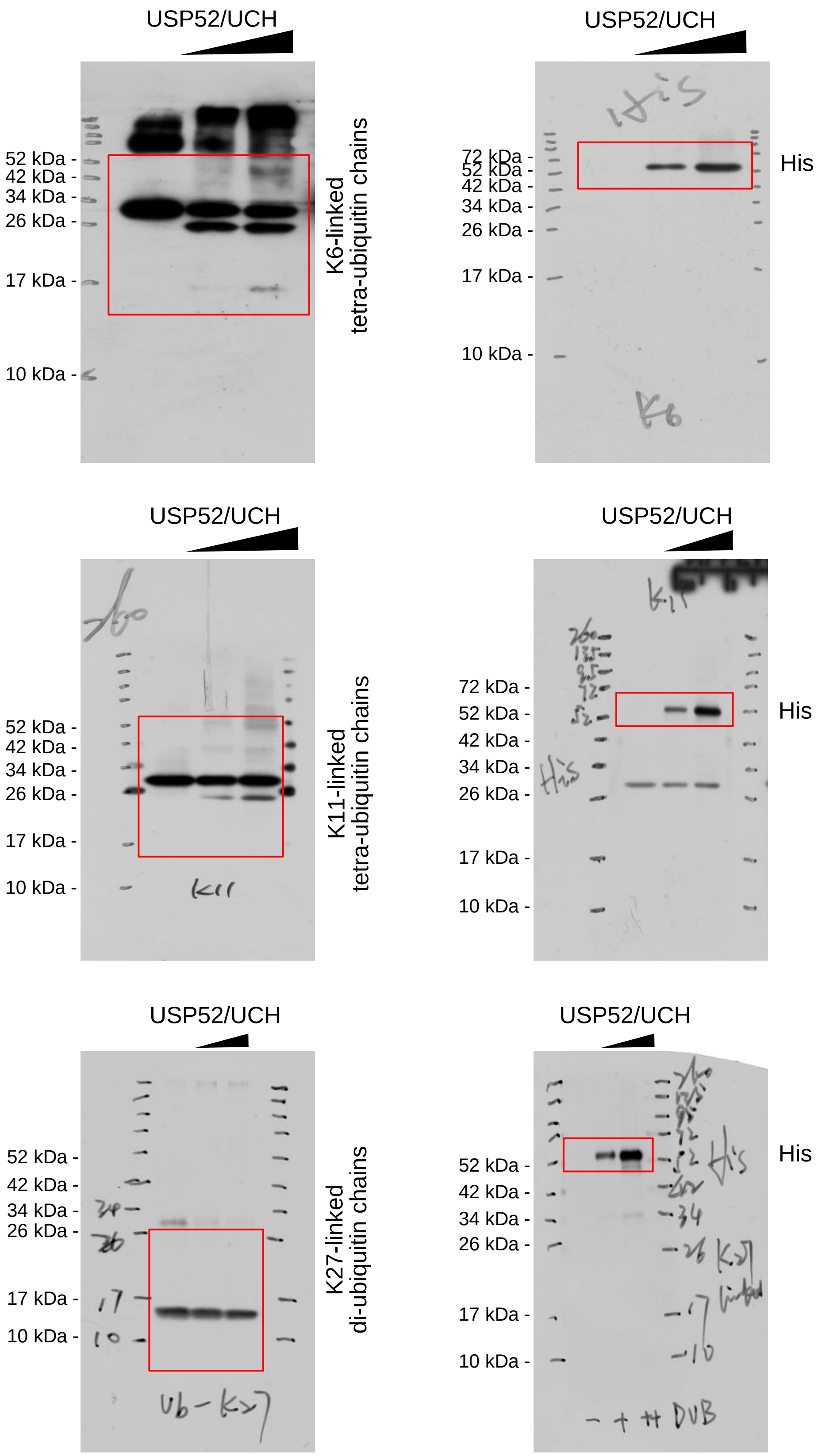

Uncropped blots related to Figure 2c

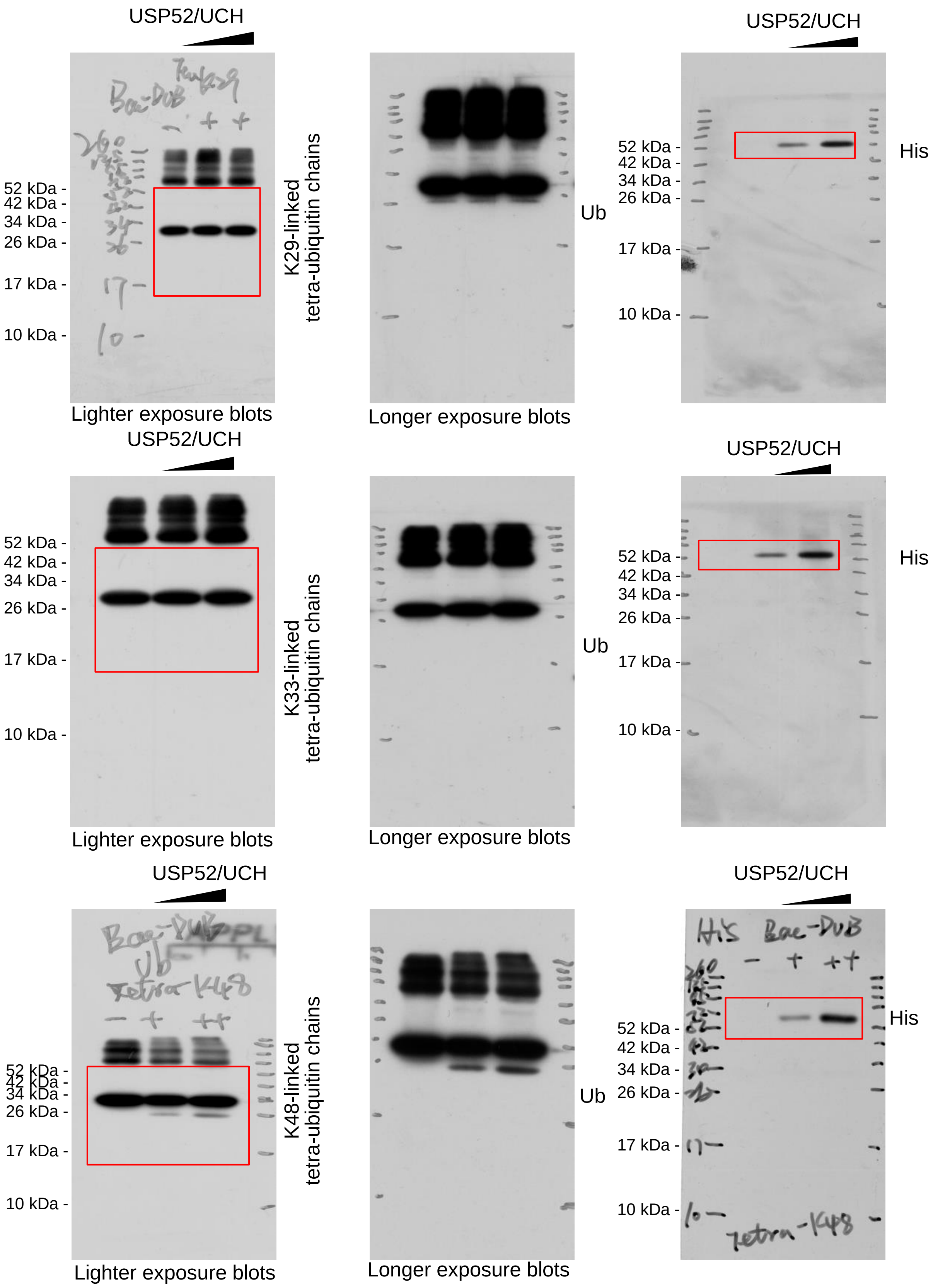

Uncropped blots related to Figure 2c

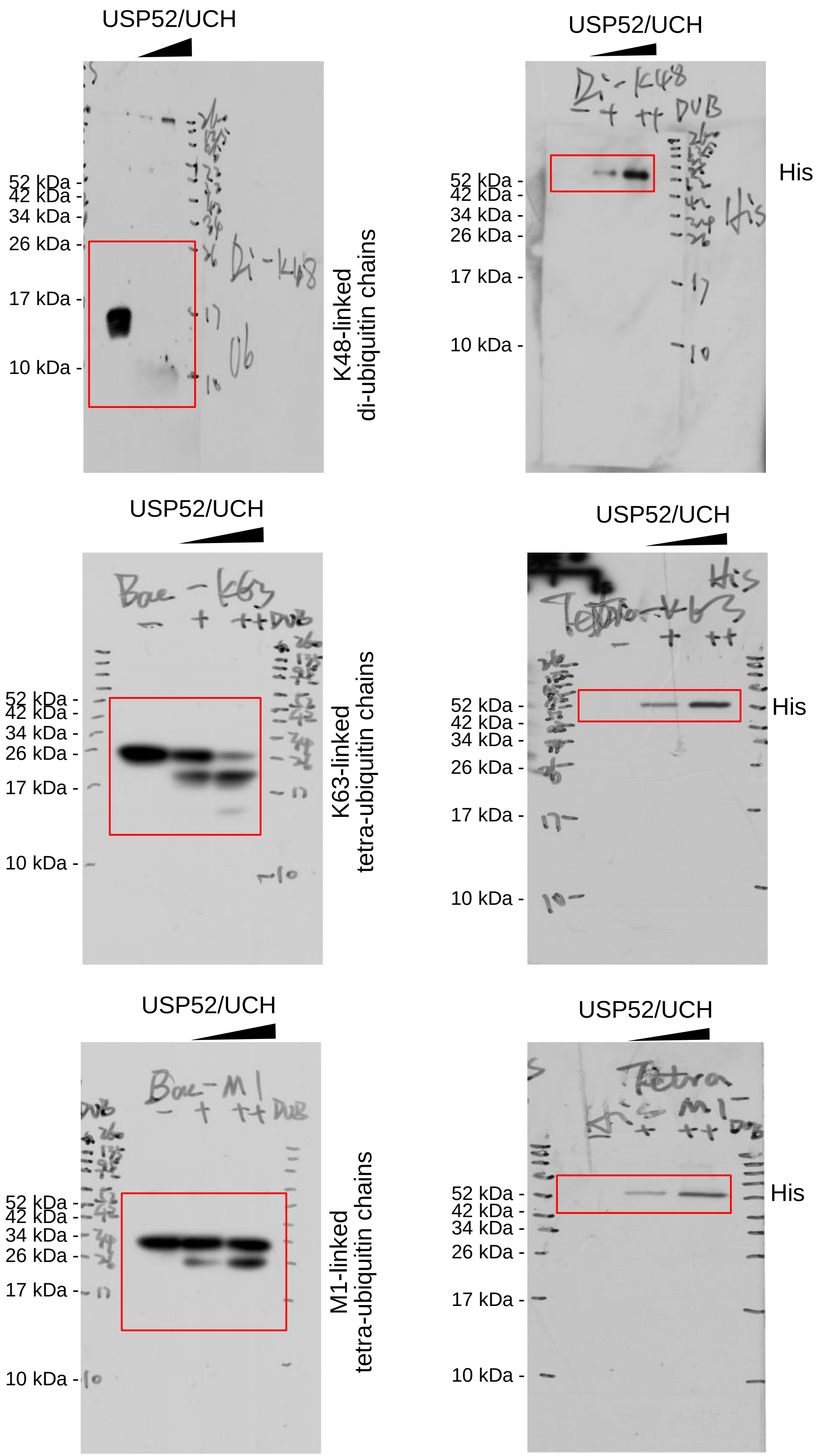

Uncropped blots related to Figure 2d

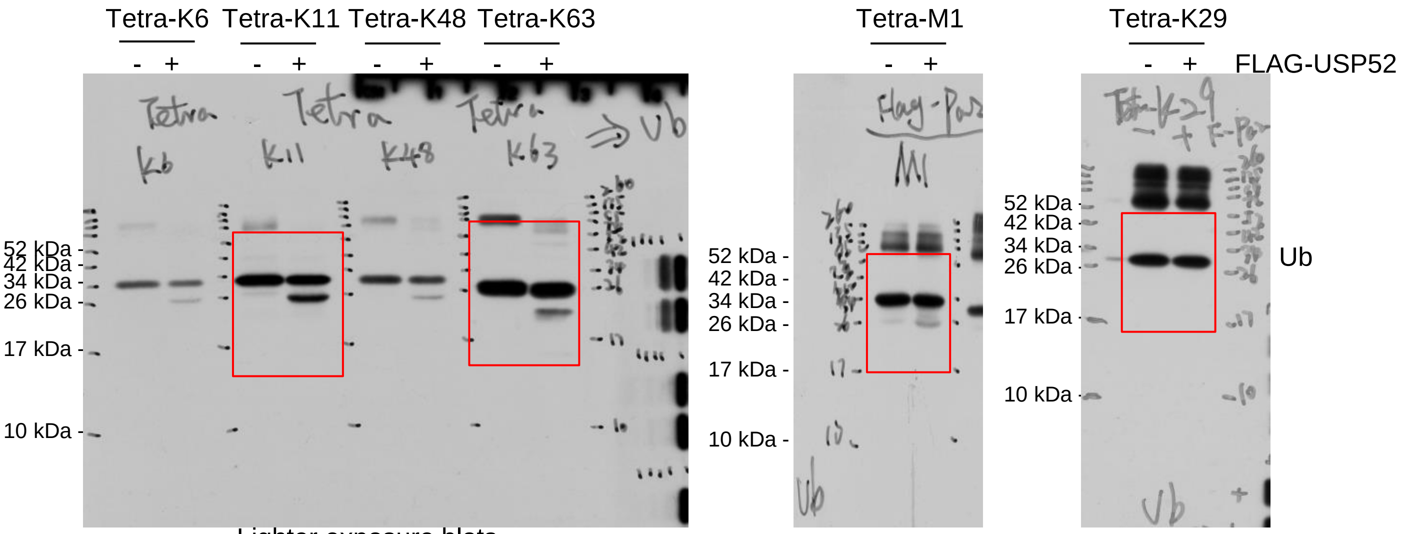

Lighter exposure blots

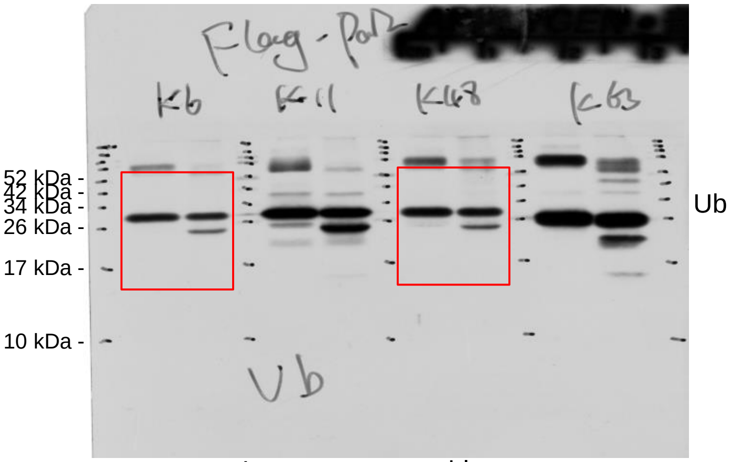

Longer exposure blots

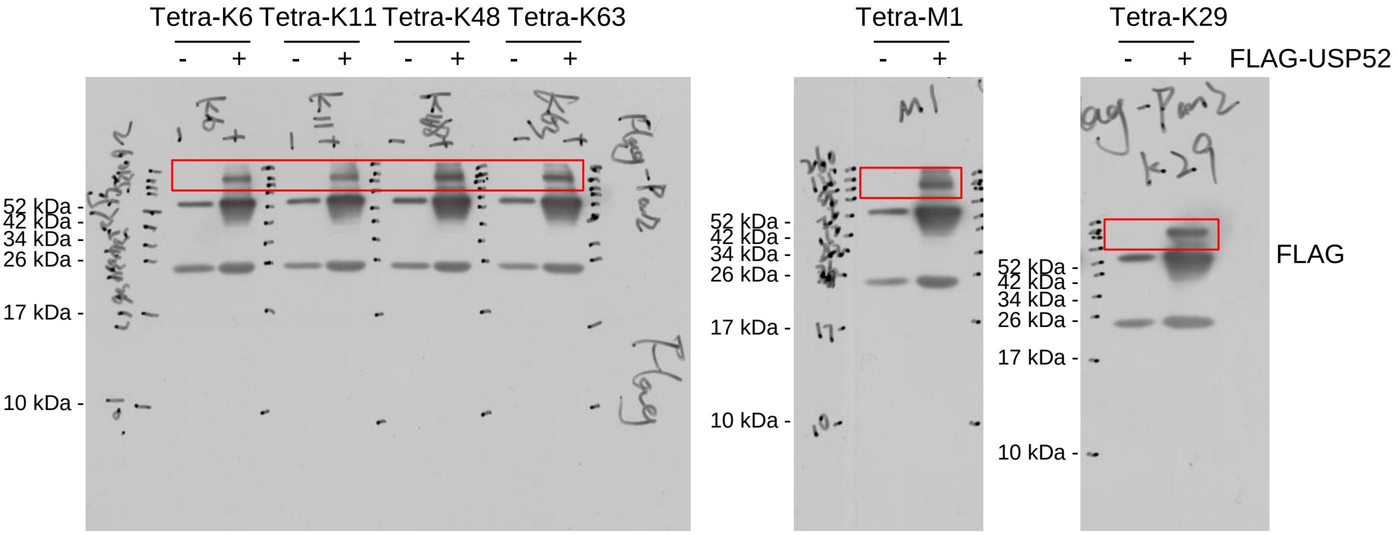

Uncropped blots related to Figure 2d

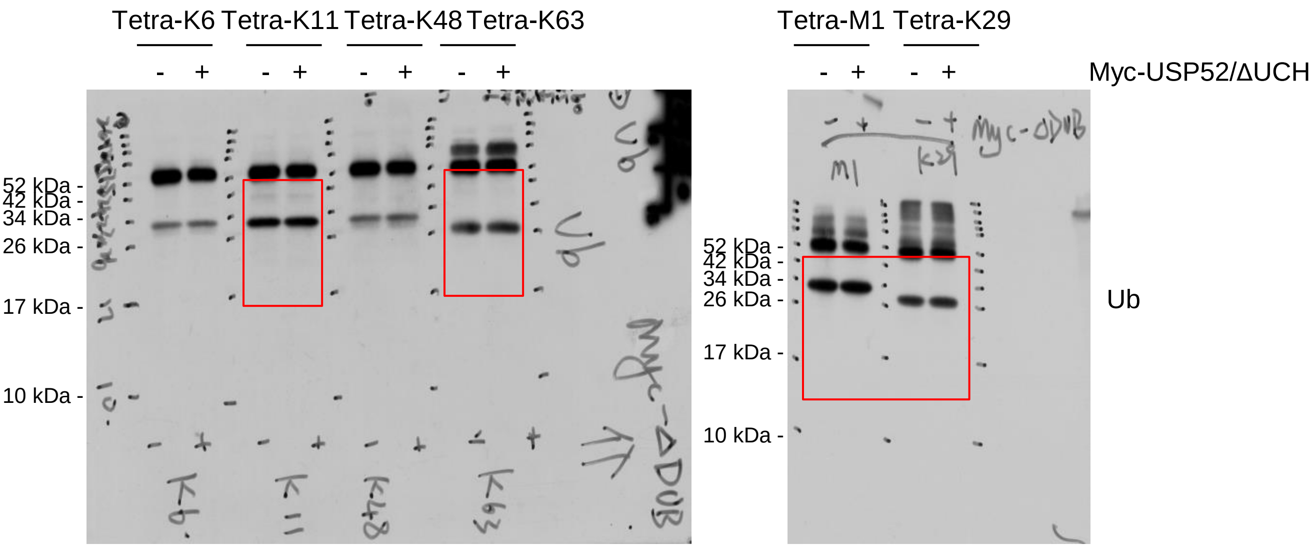

Lighter exposure blots

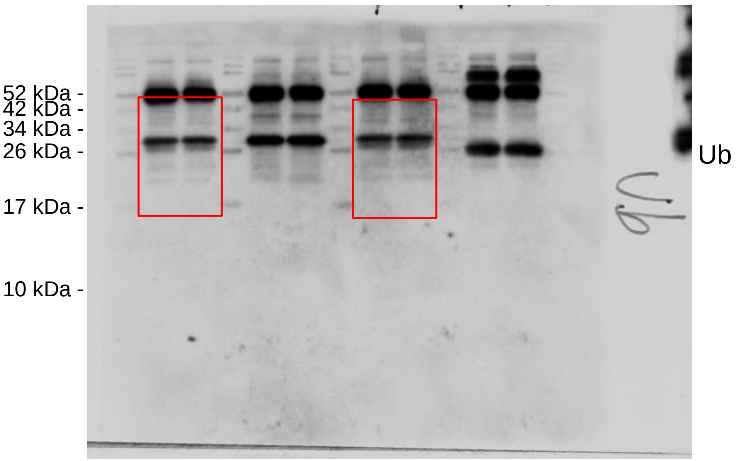

Longer exposure blots

Uncropped blots related to Figure 2d

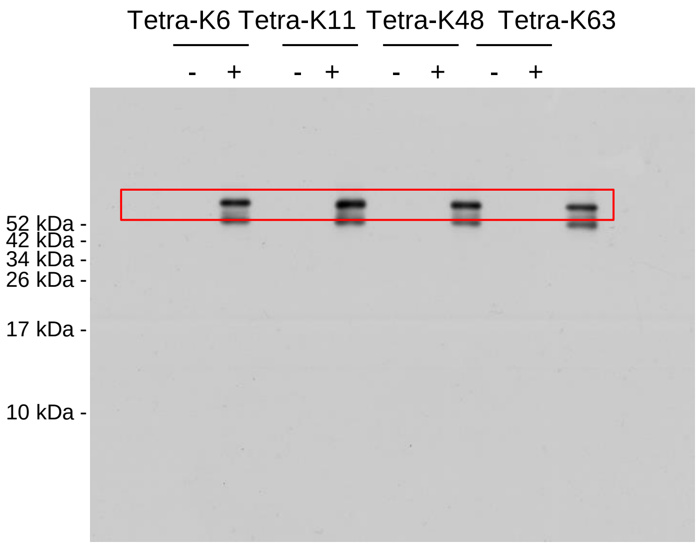

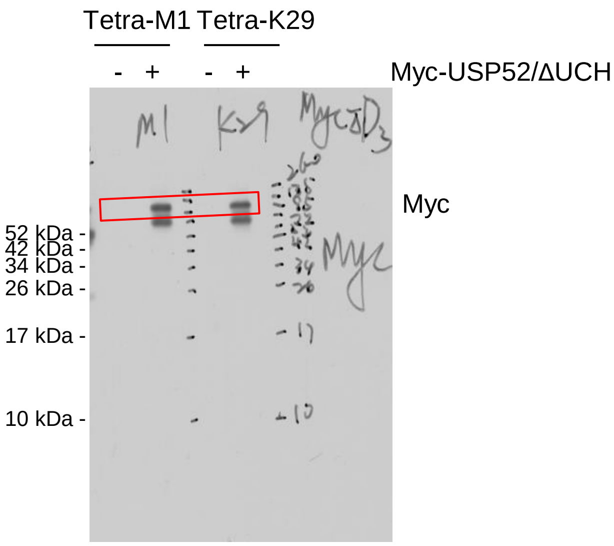

Longer exposure blots

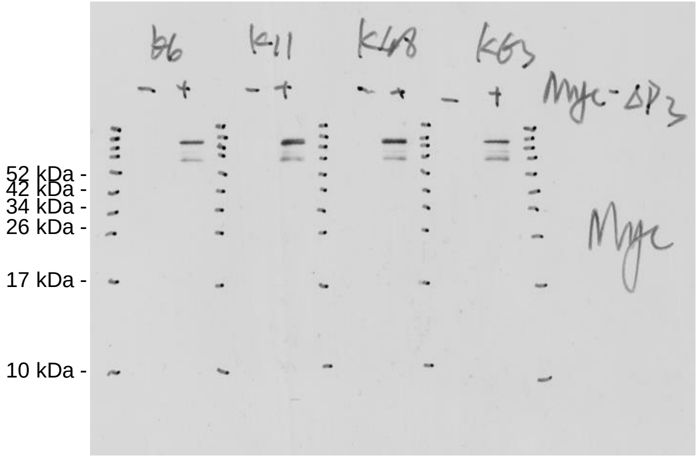

Lighter exposure blots

Uncropped blots related to Figure 2e

+	–	–	–	–	Vector
–	+	+	–	–	His-USP52/UCH
–	–	–	+	+	His-USP7-C
–	–	+	–	+	NEM
+	+	+	+	+	K48-linked Ubs

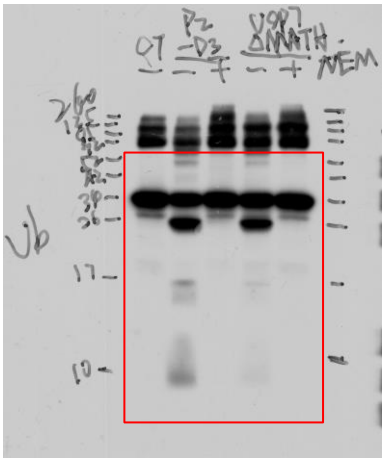

(USP7-ΔMATH: USP7-C)

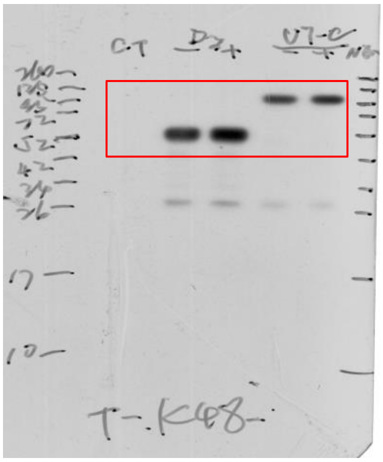

Uncropped blots related to Figure 2e

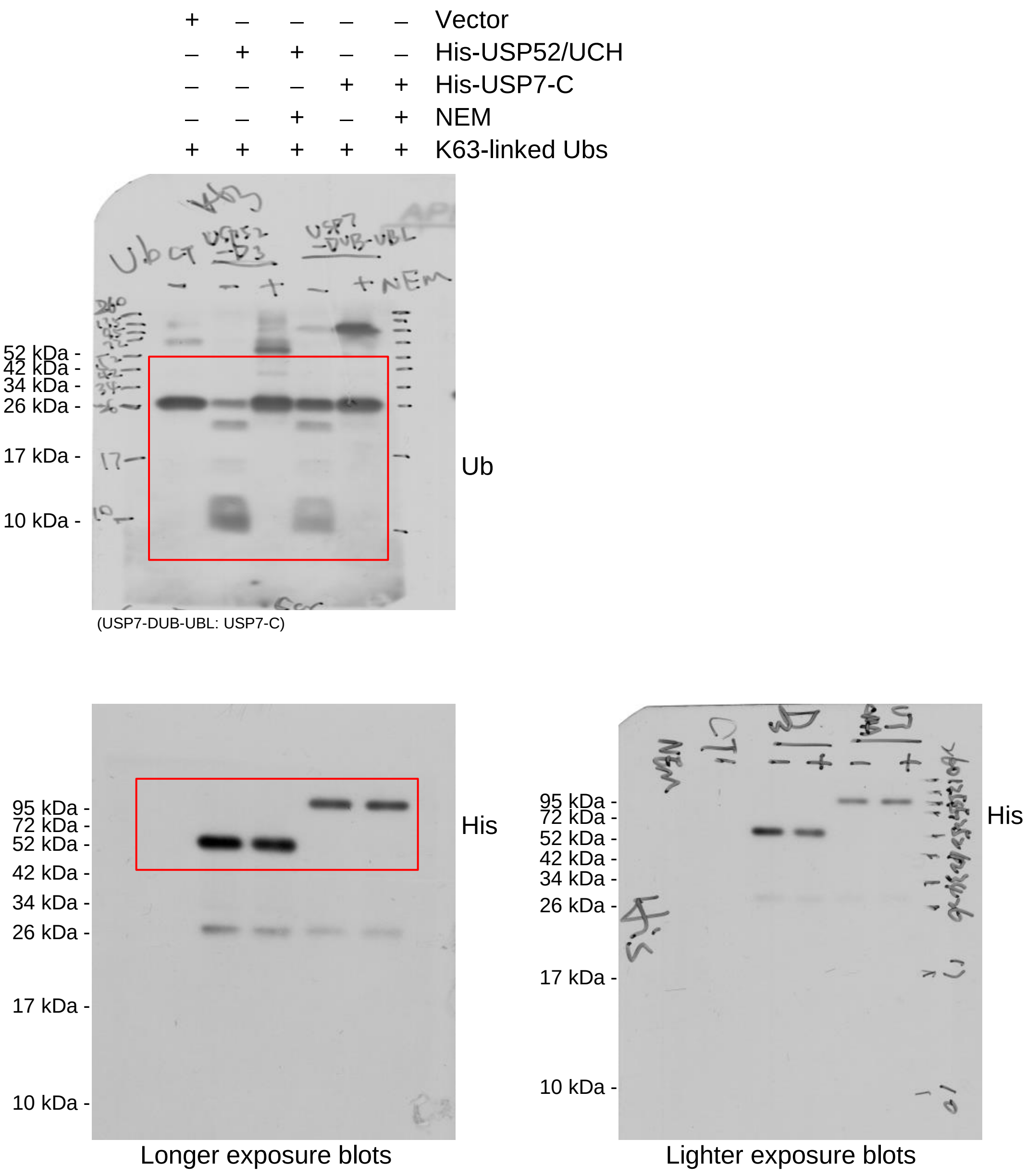

Uncropped blots related to Figure 2f

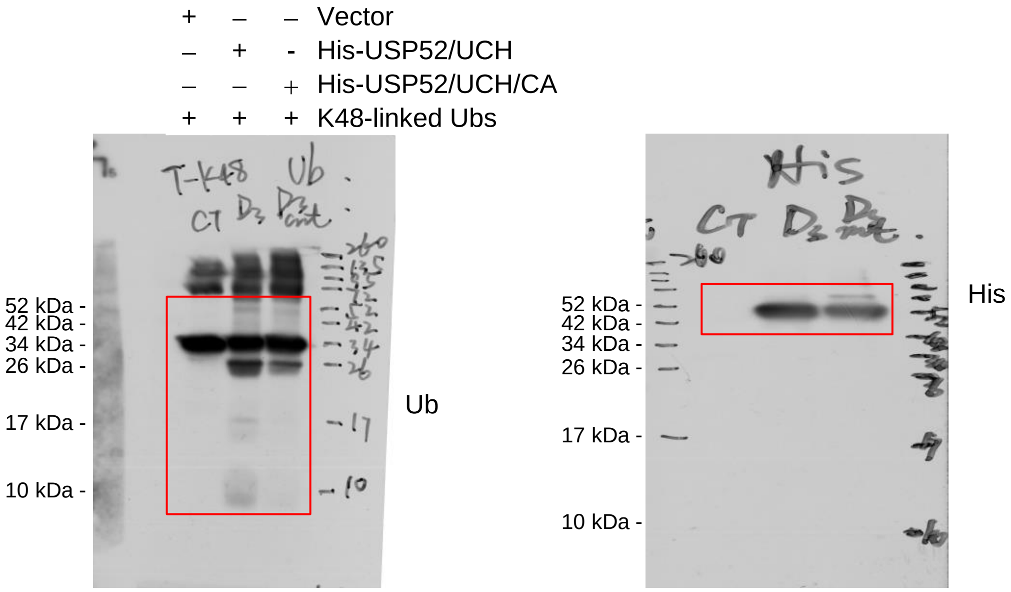

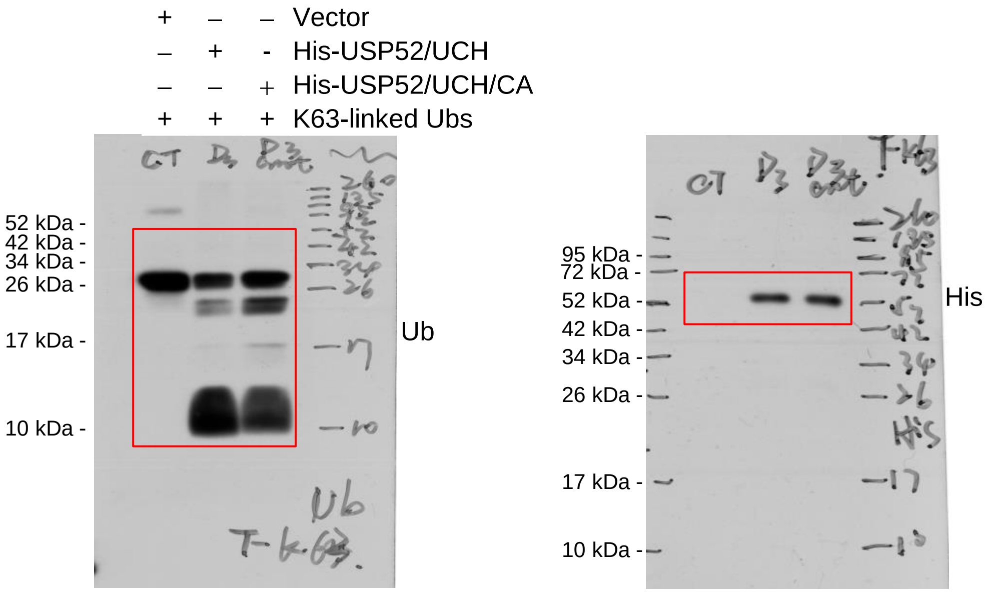

Uncropped blots related to Figure 3a

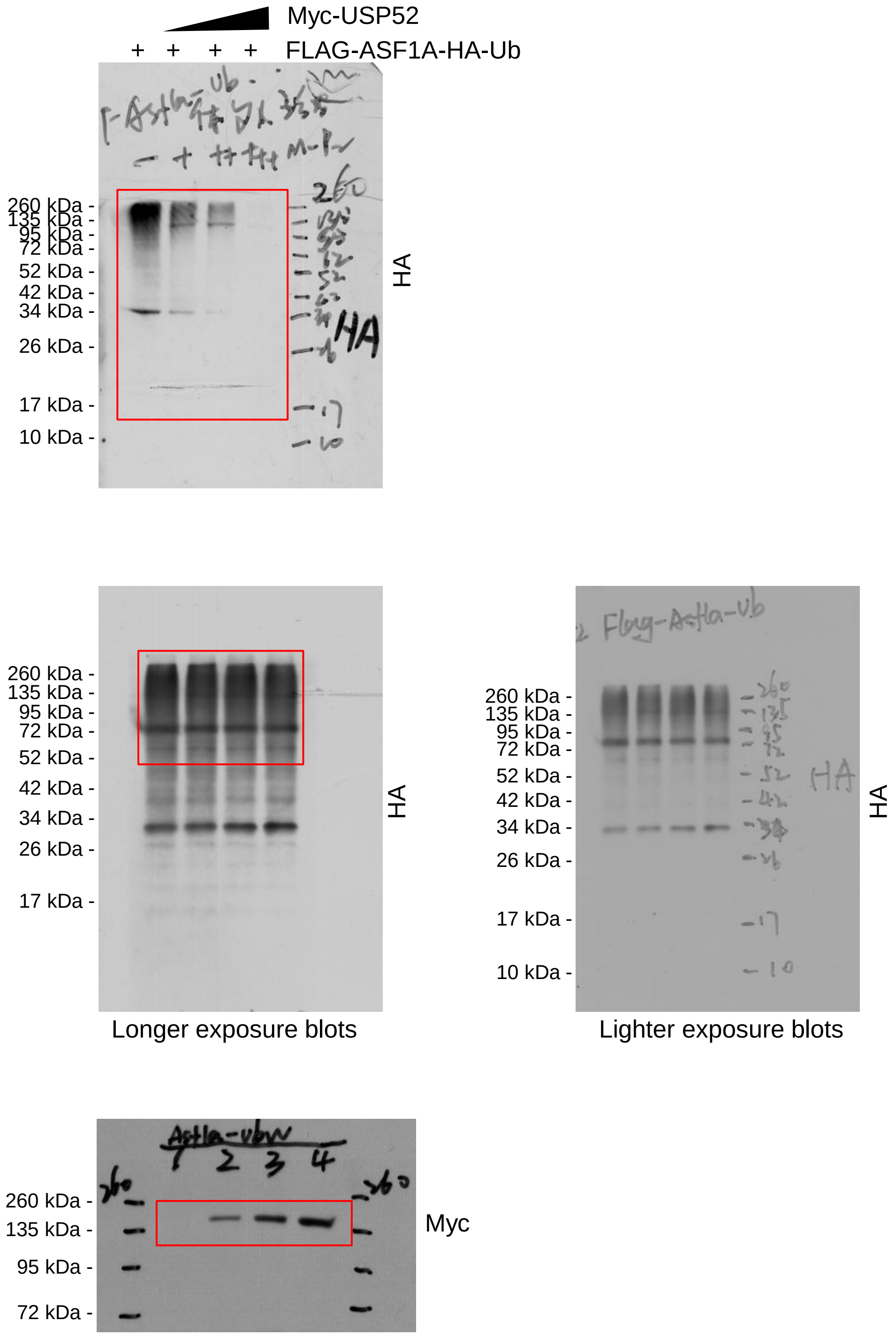

Uncropped blots related to Figure 3b

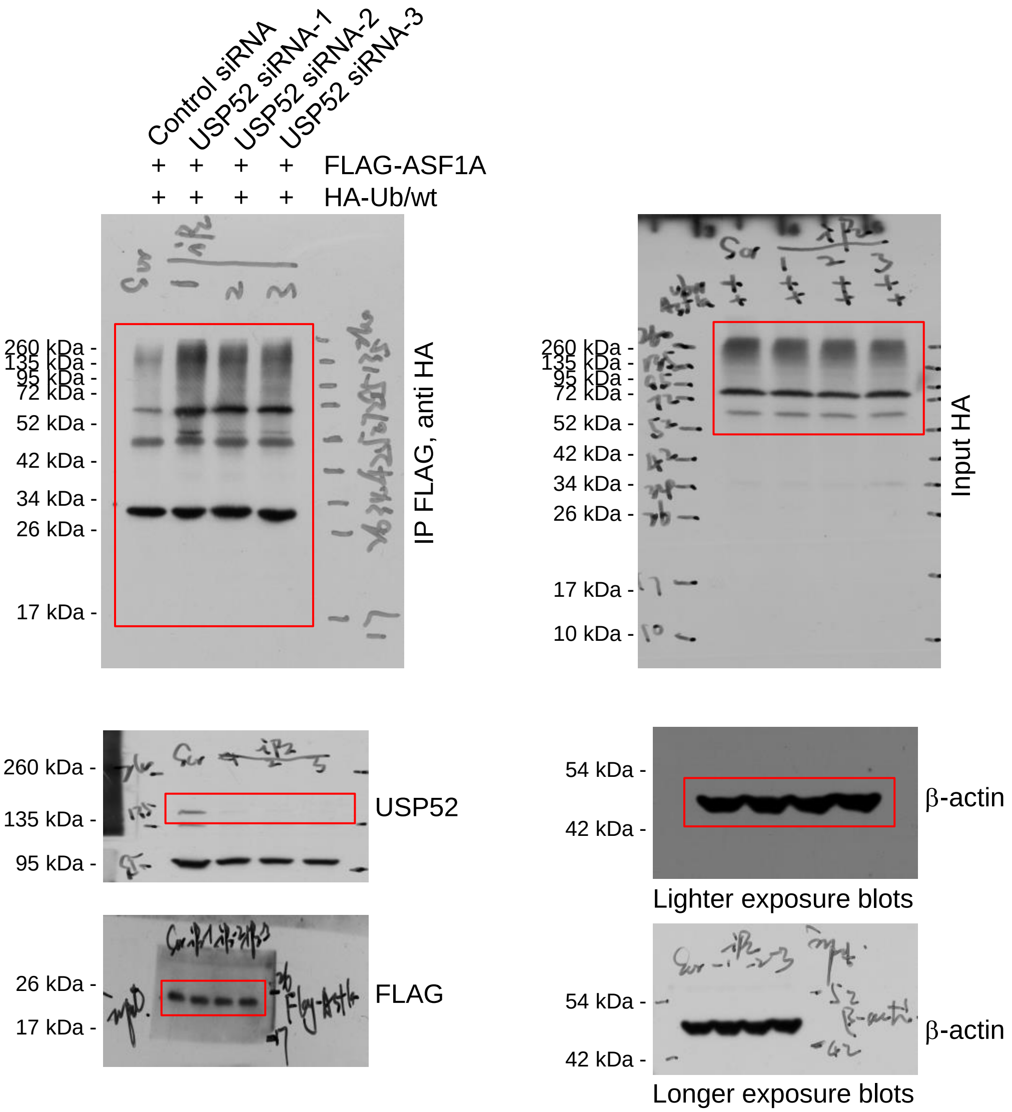

Uncropped blots related to Figure 3c

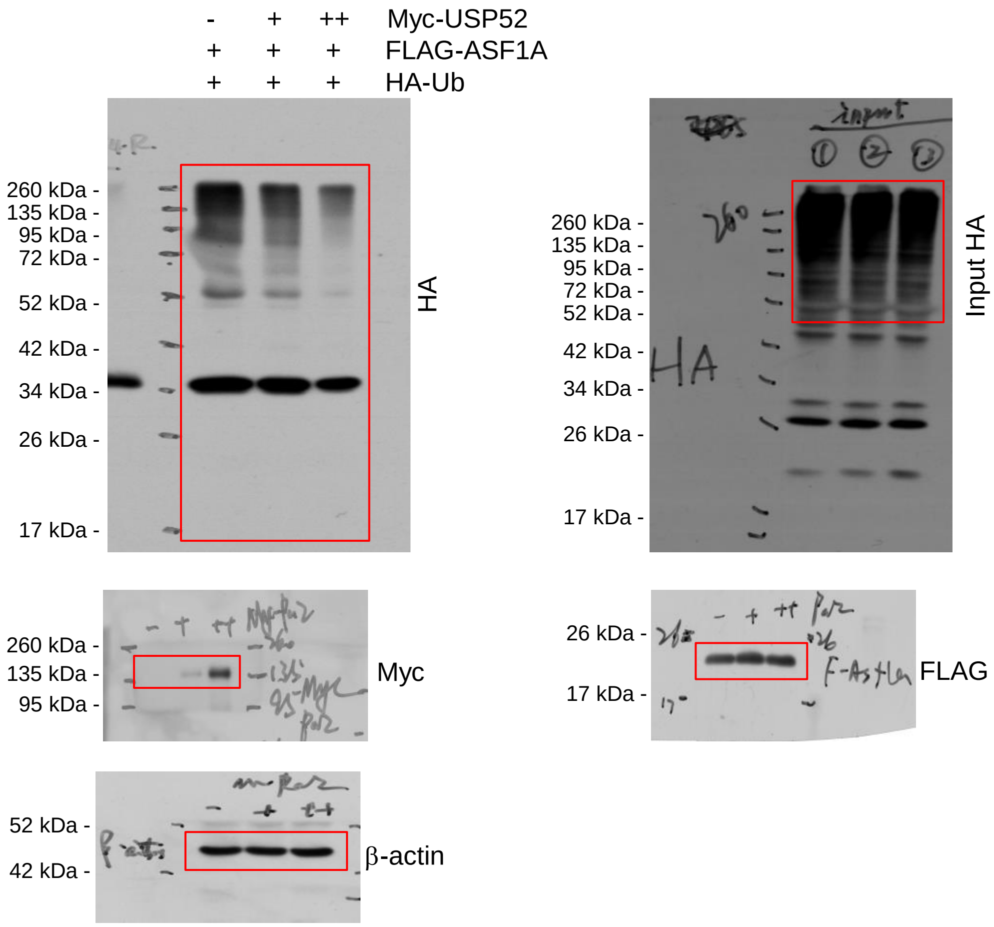

Uncropped blots related to Figure 3d

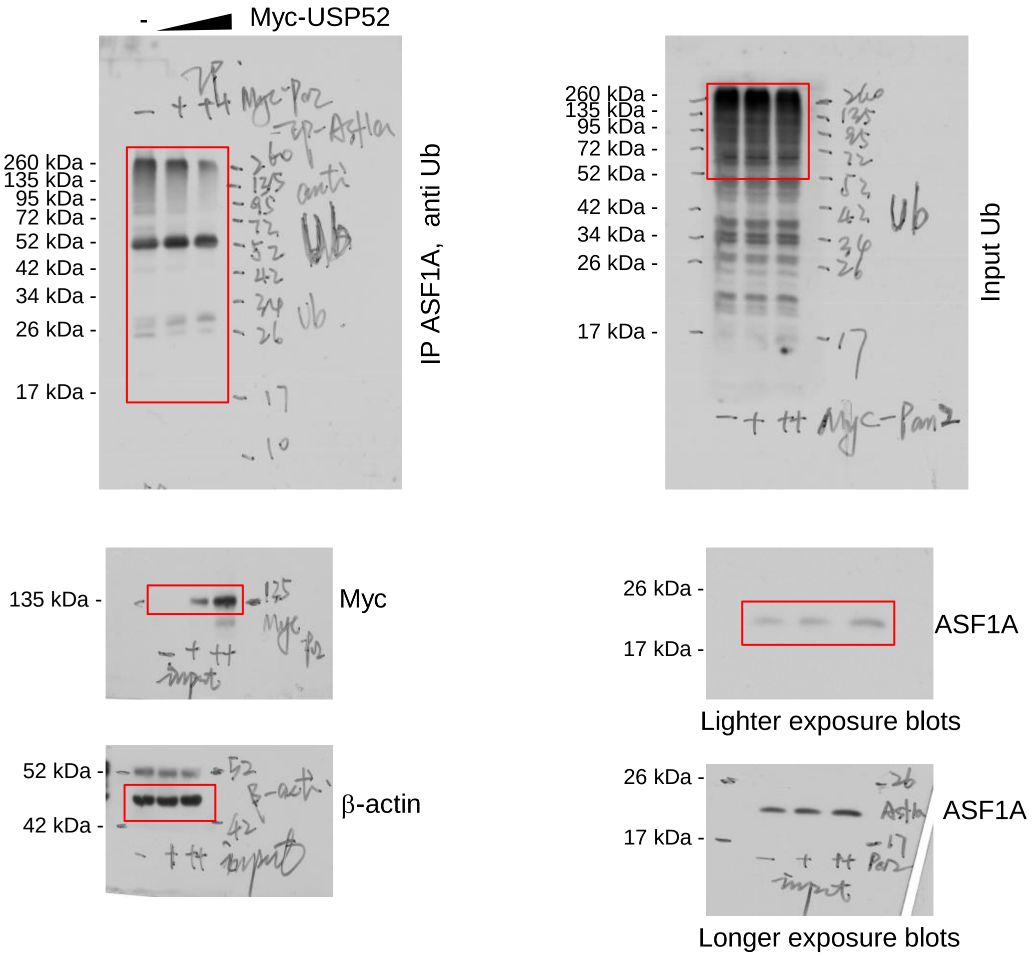

Uncropped blots related to Figure 3e

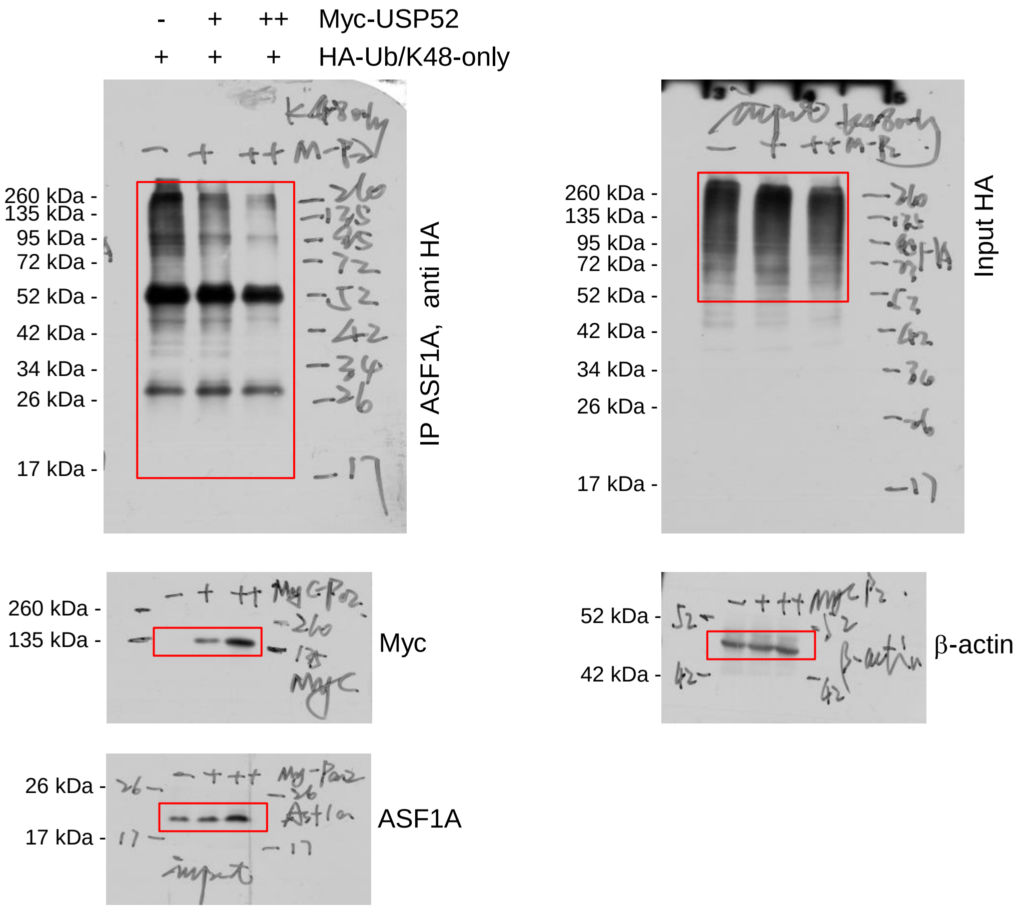

Uncropped blots related to Figure 3f

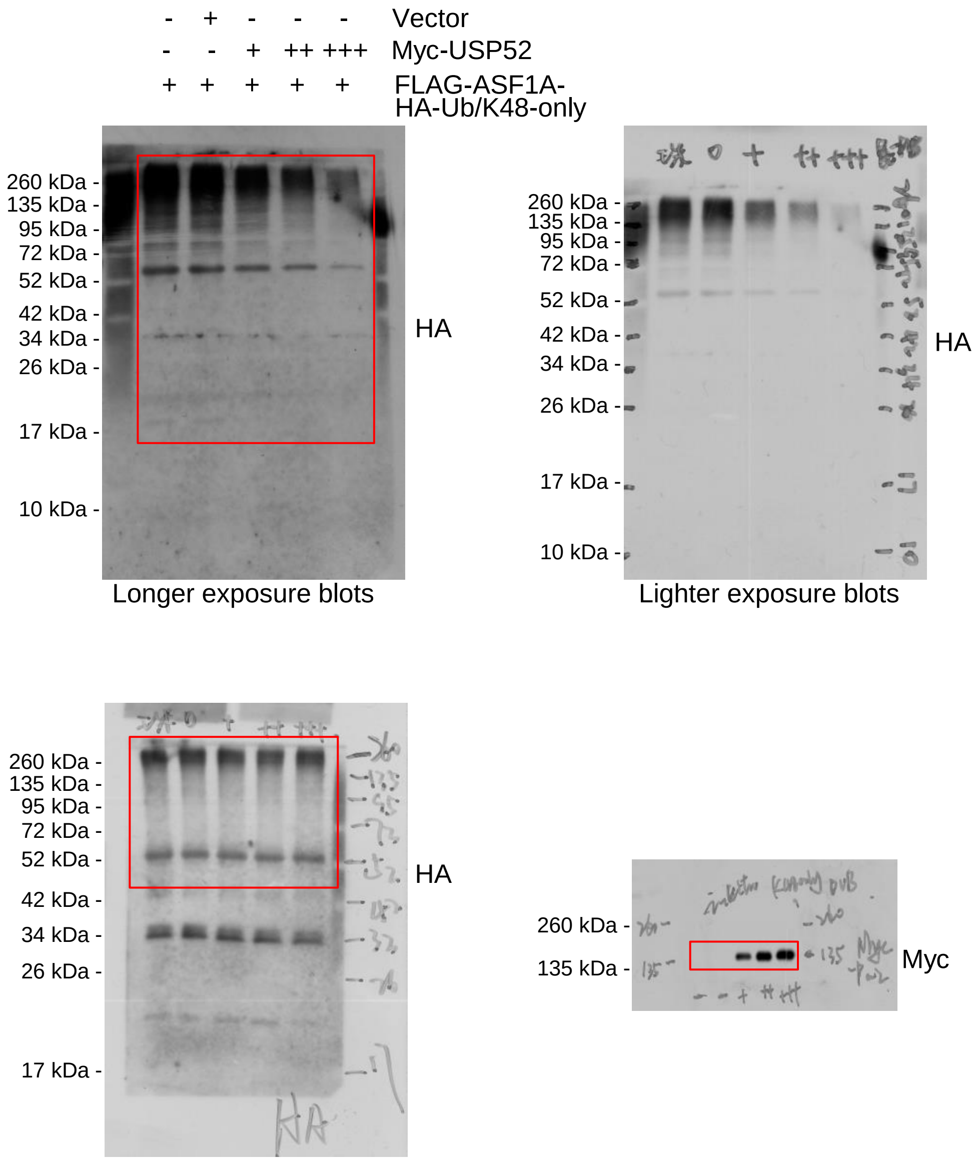

Uncropped blots related to Figure 3g

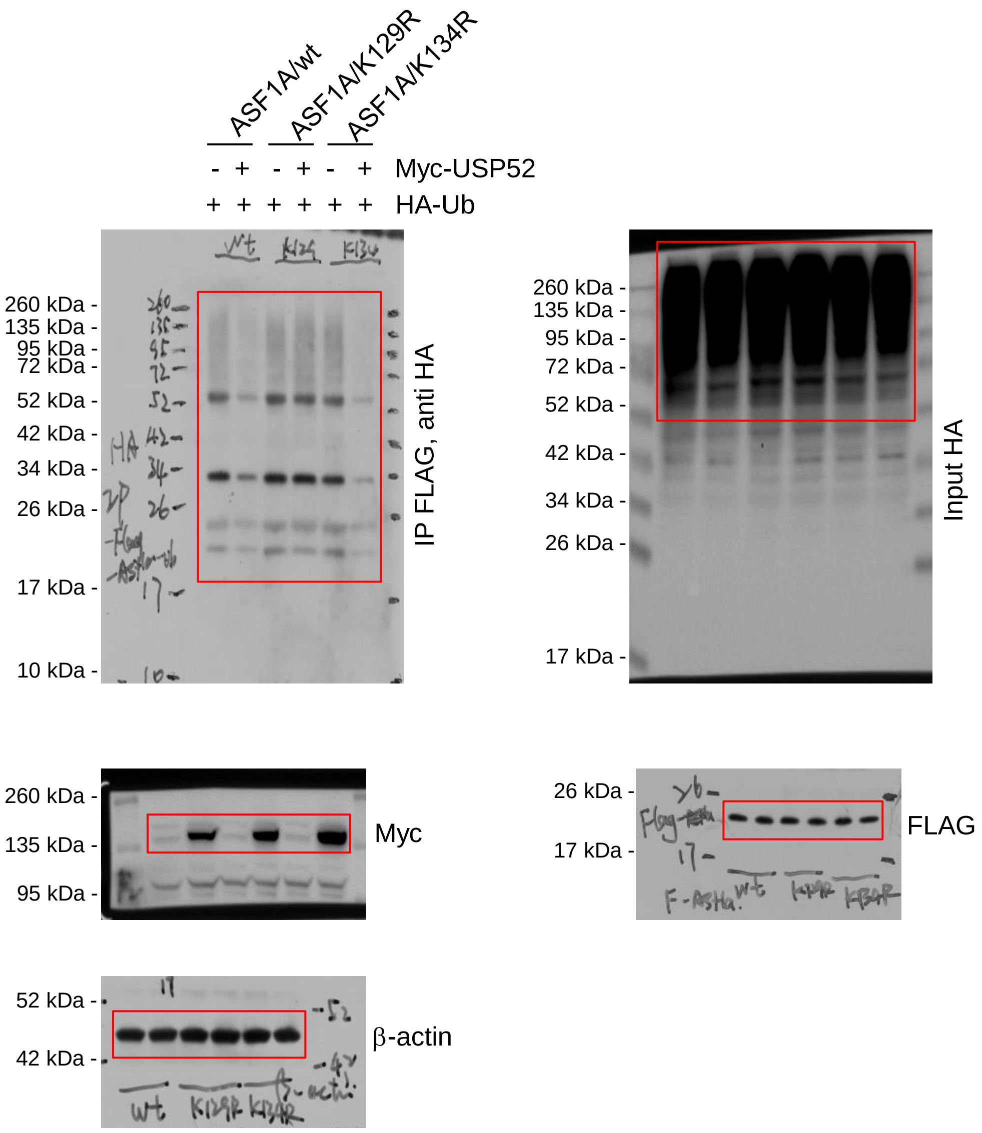

Uncropped blots related to Figure 3g

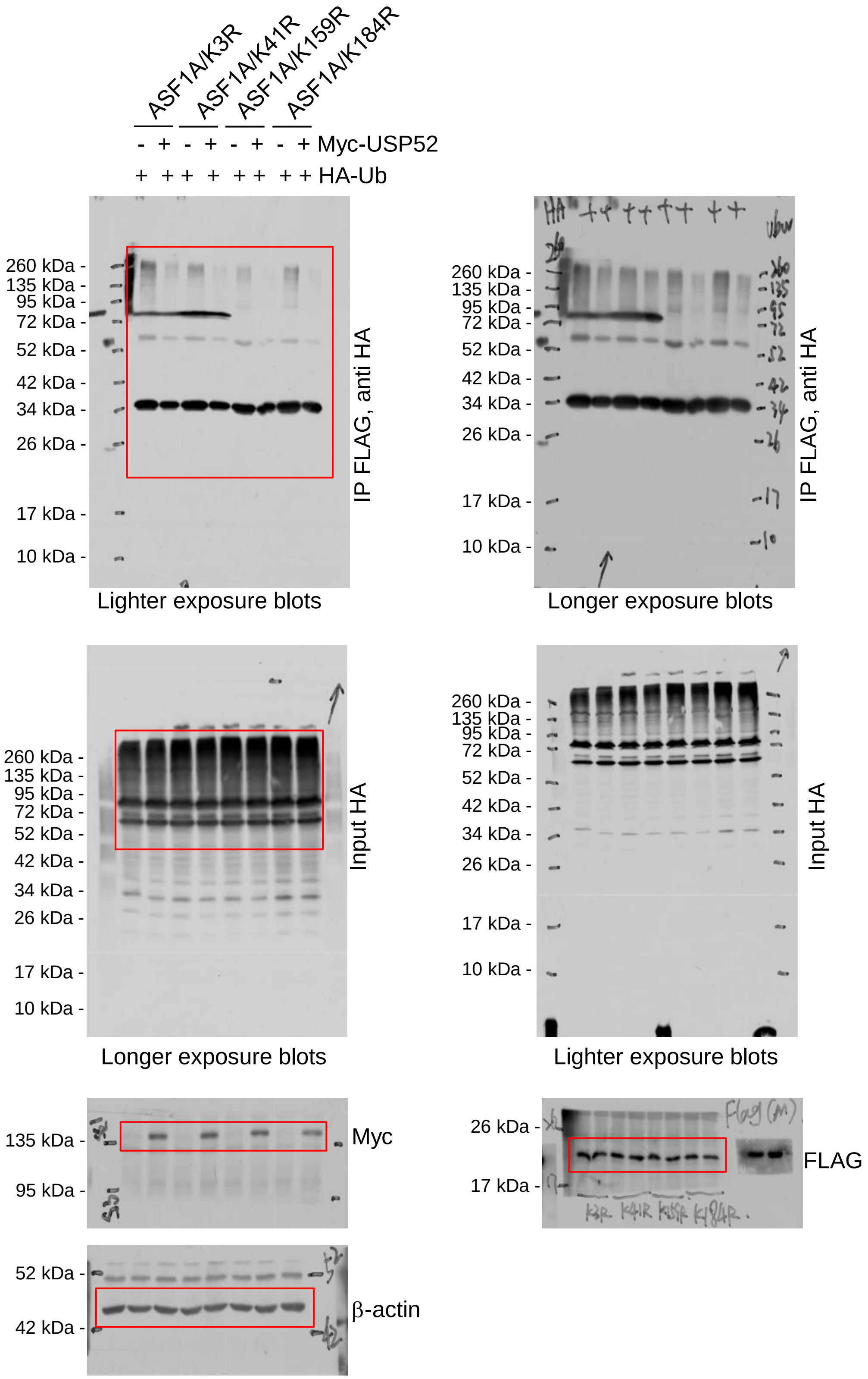

Uncropped blots related to Figure 4a

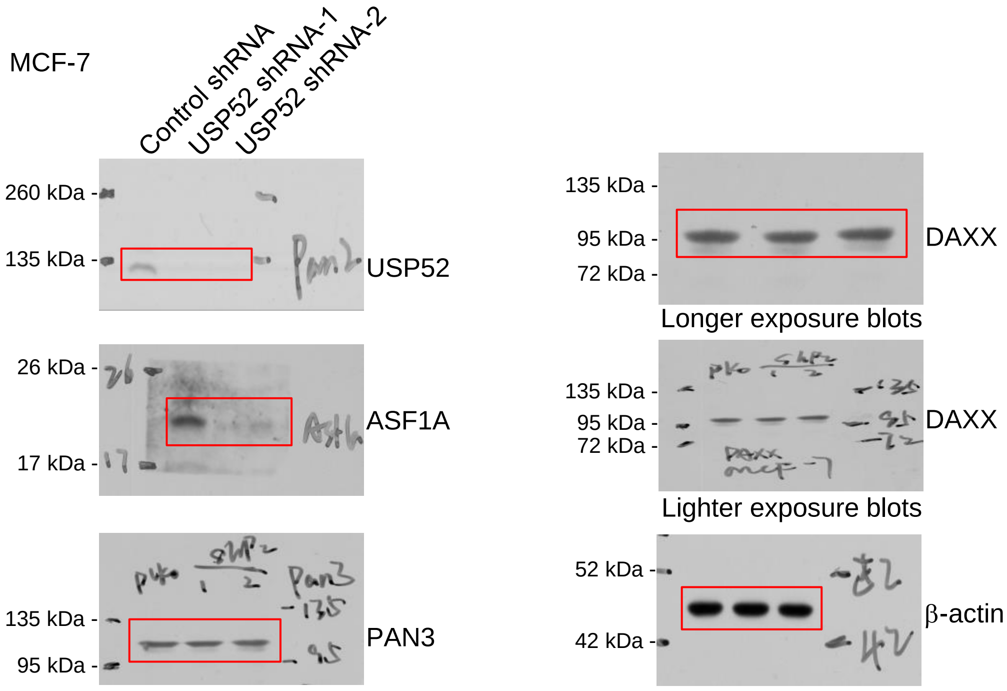

Uncropped blots related to Figure 4b

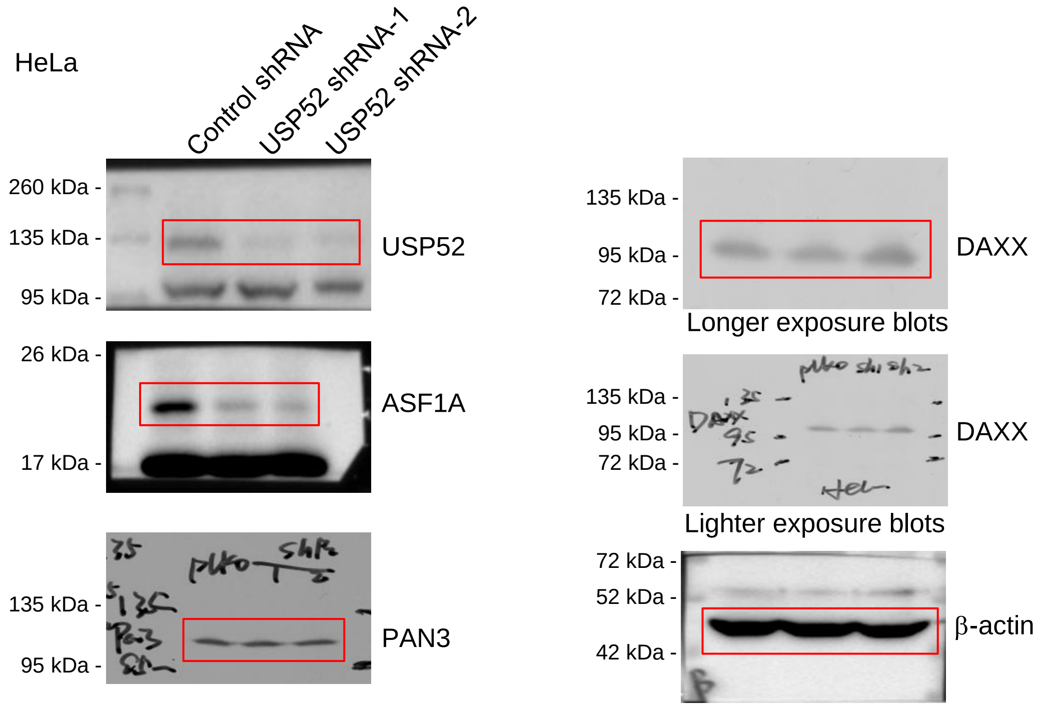

Uncropped blots related to Figure 4c

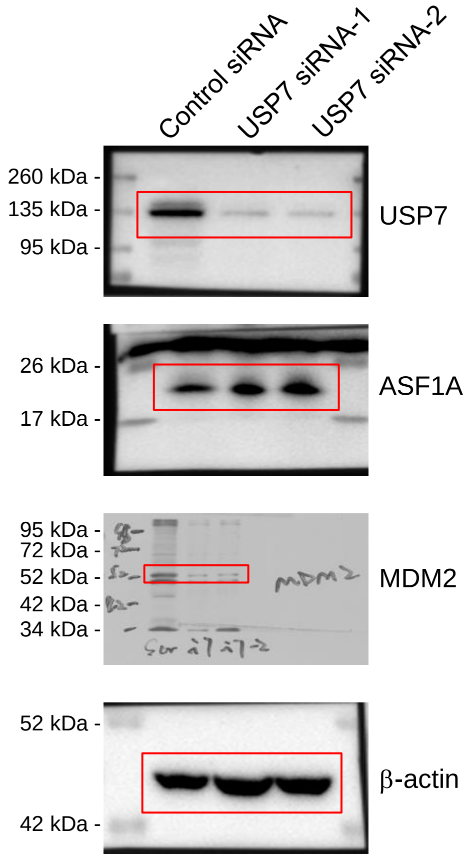

Uncropped blots related to Figure 4d

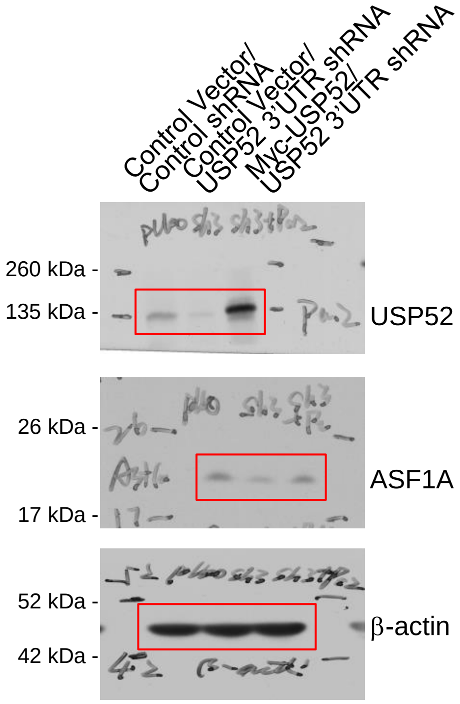

Uncropped blots related to Figure 4e

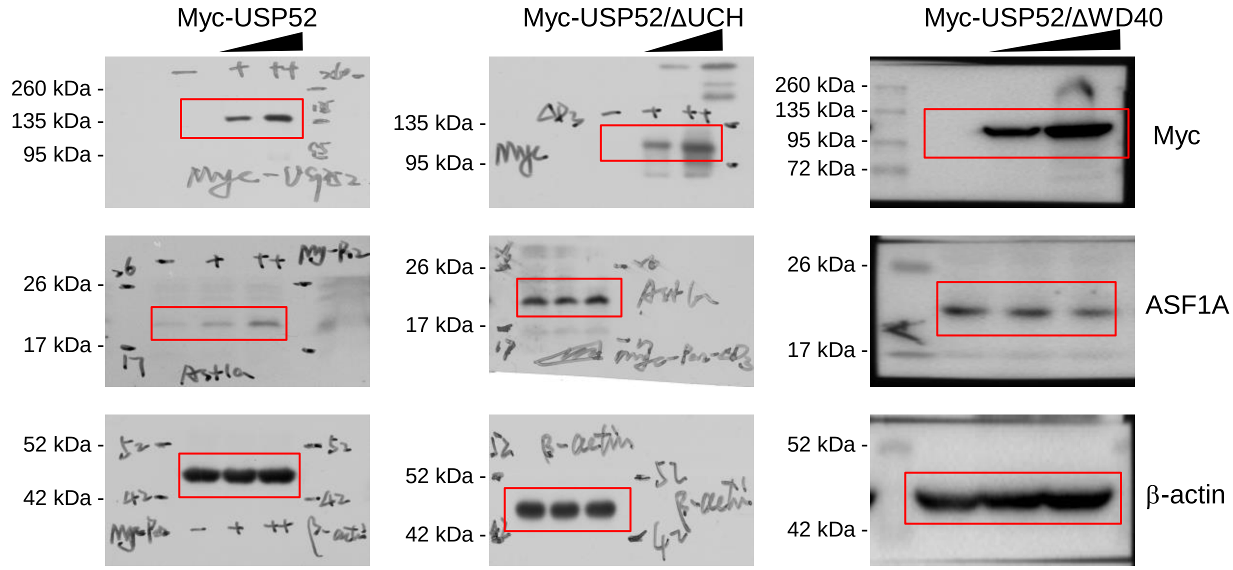

Uncropped blots related to Figure 4f

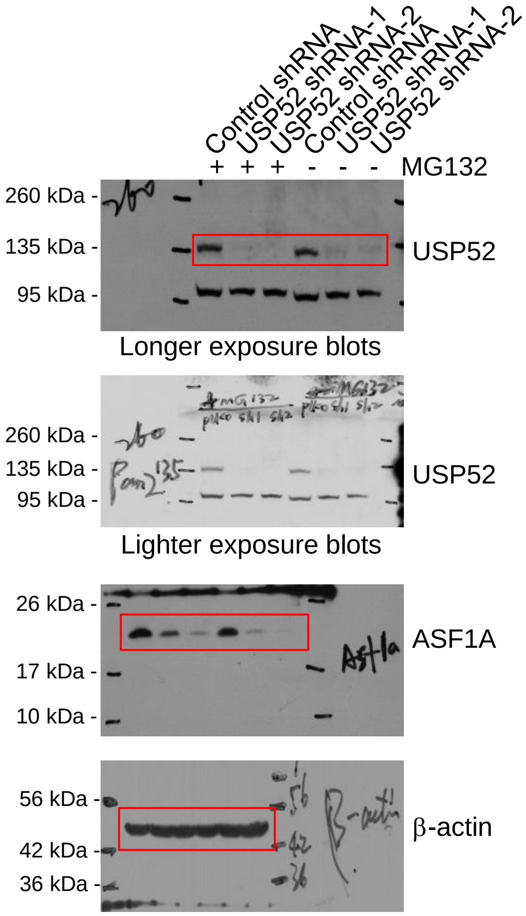

Uncropped blots related to Figure 4g

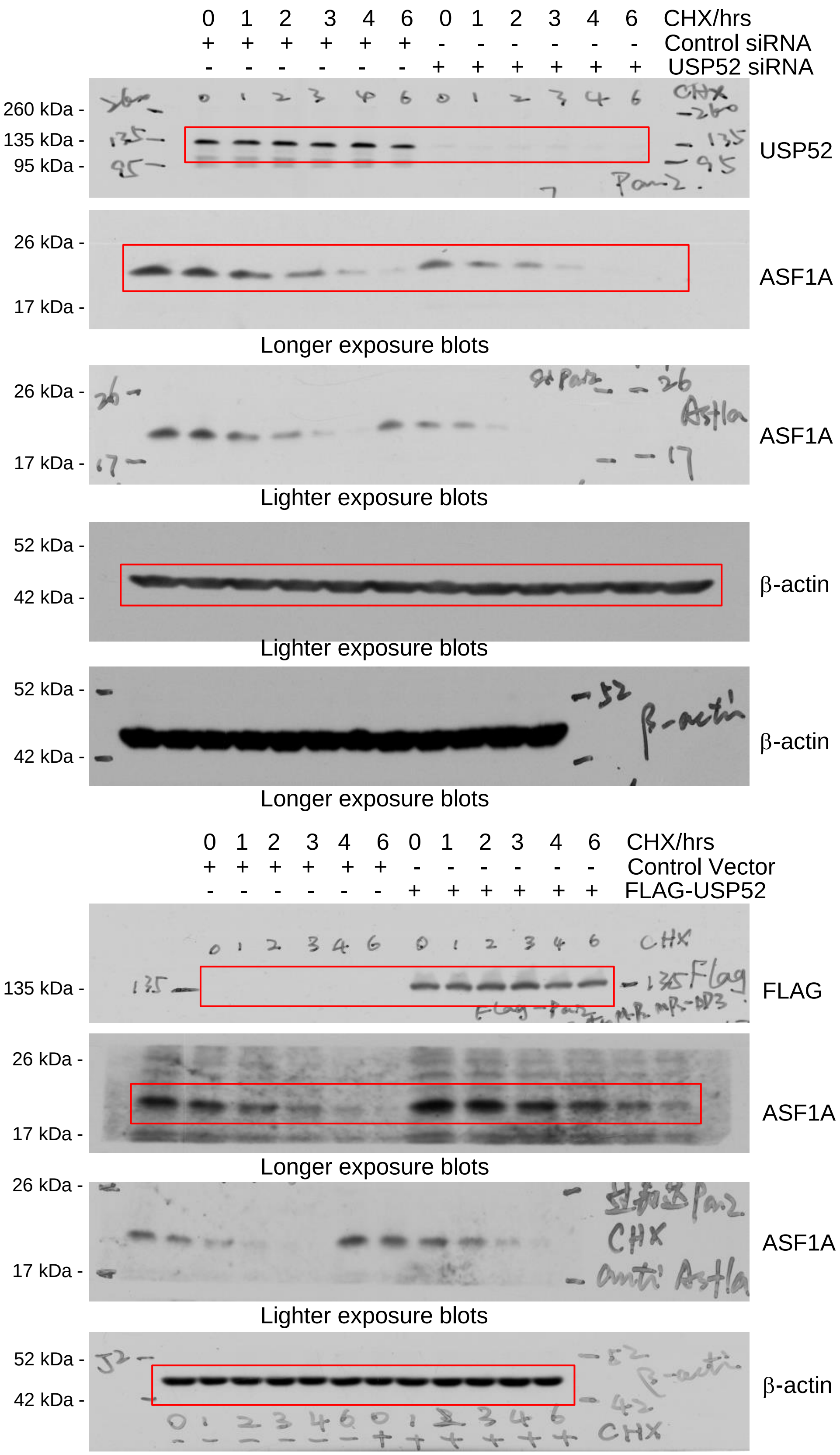

Uncropped blots related to Figure 4h

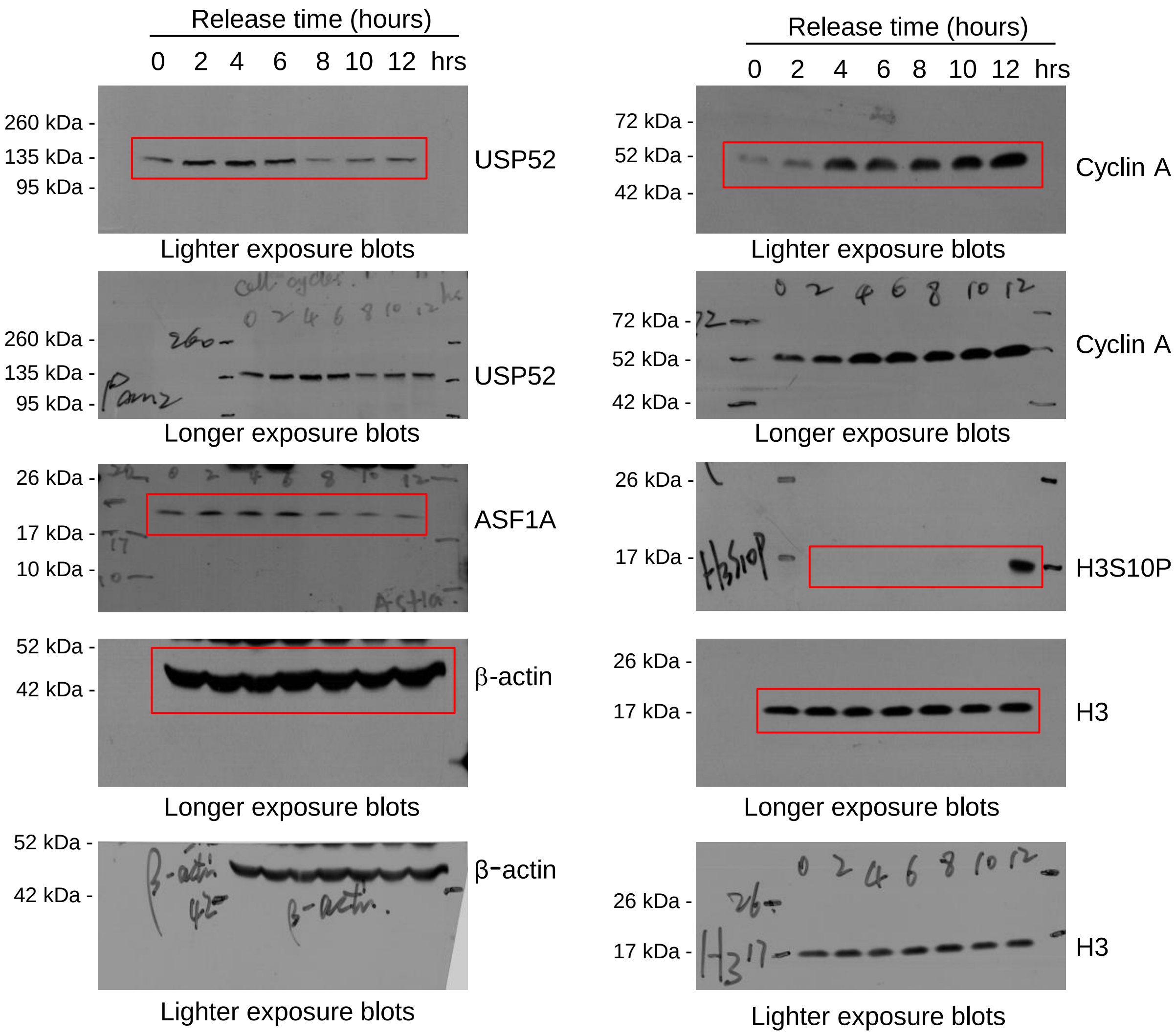

Uncropped blots related to Figure 4i

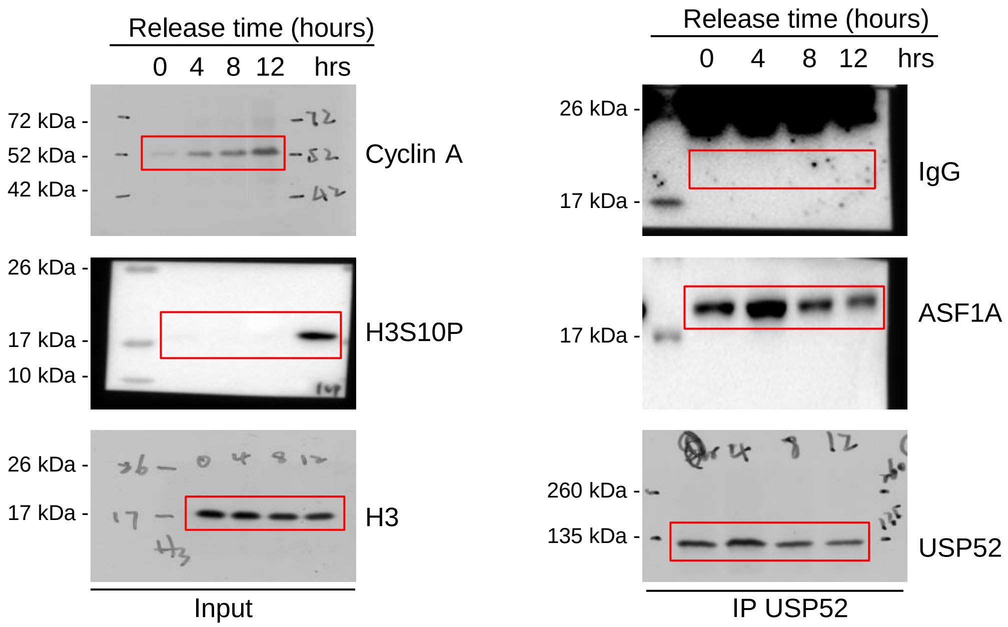

Uncropped blots related to Figure 4j

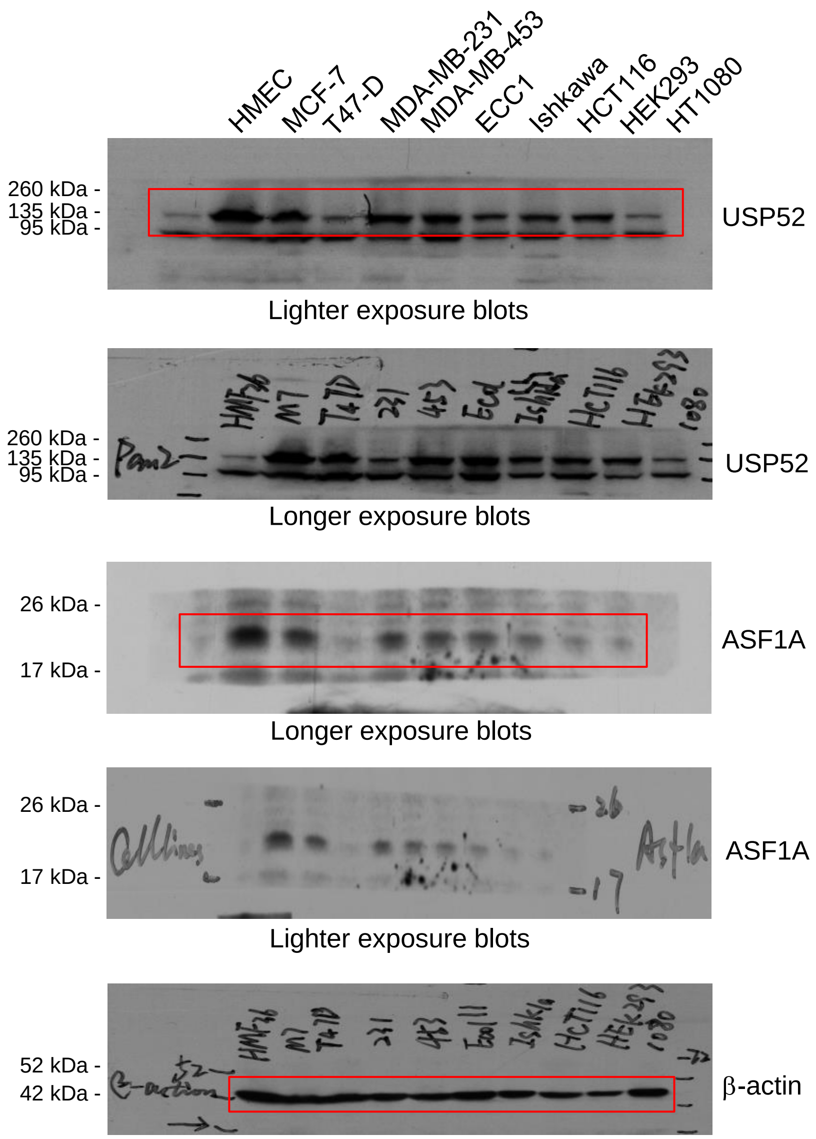

Uncropped blots related to Figure 5b

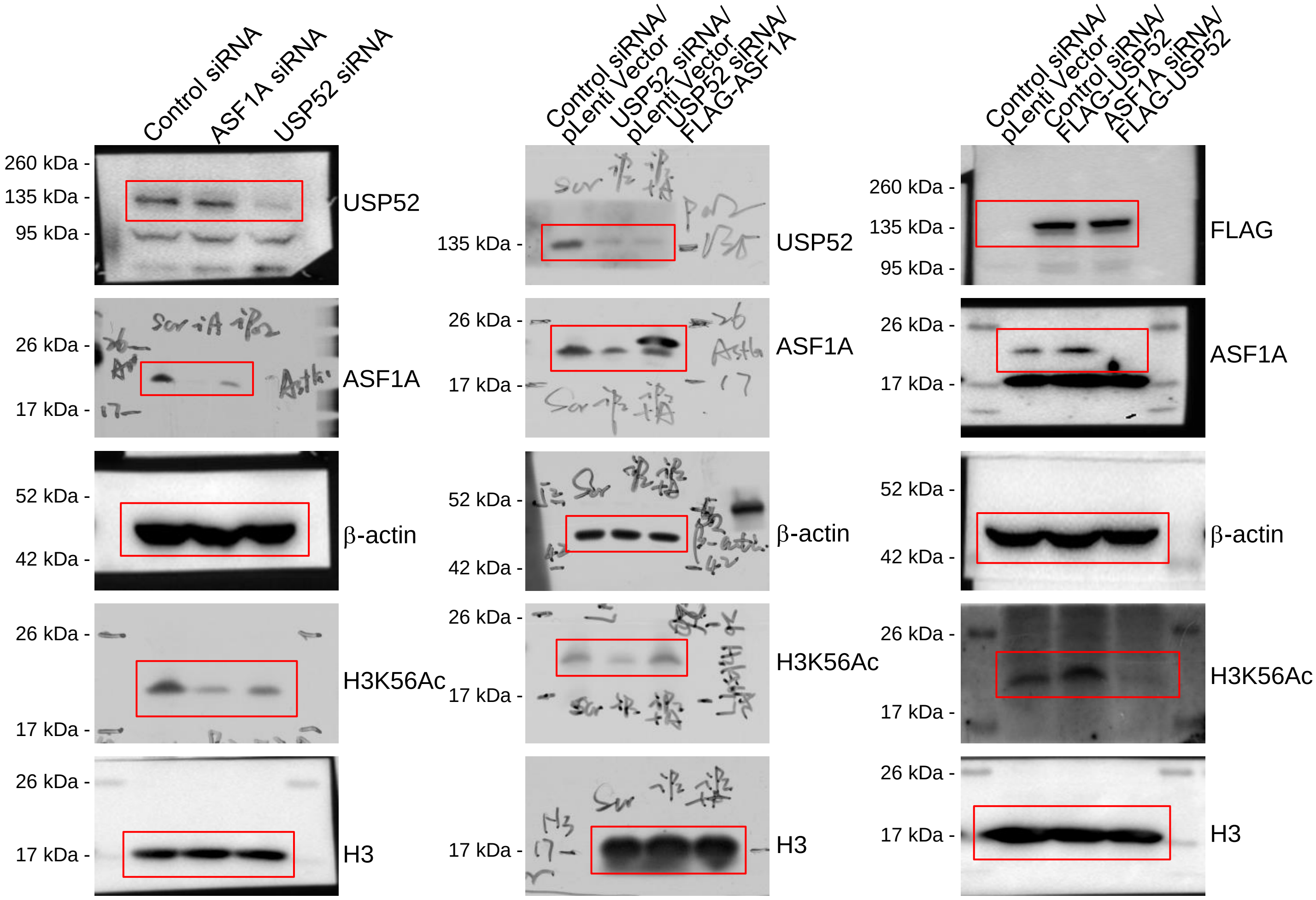

Uncropped blots related to Figure 5b

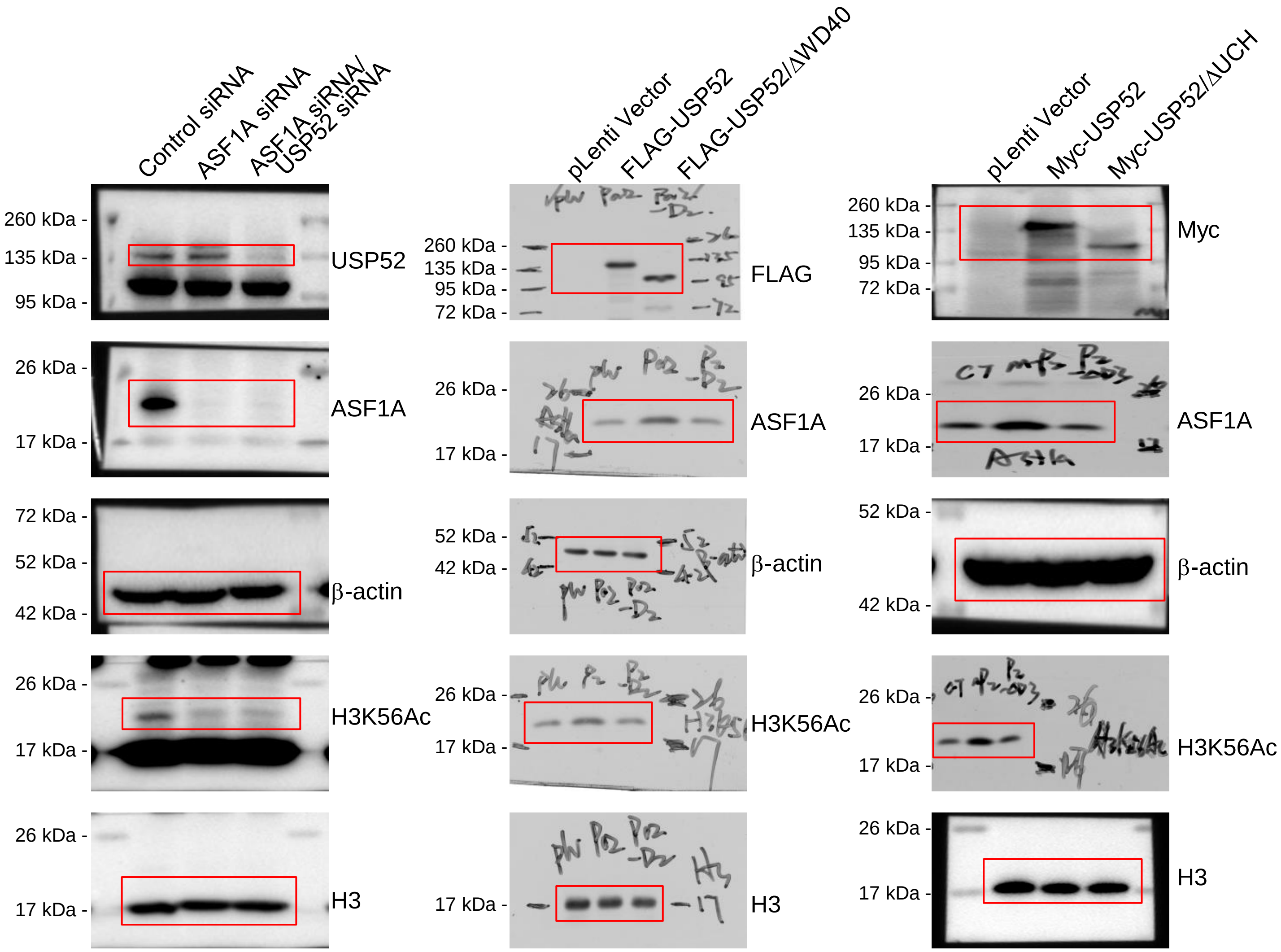

Uncropped blots related to Figure 5e

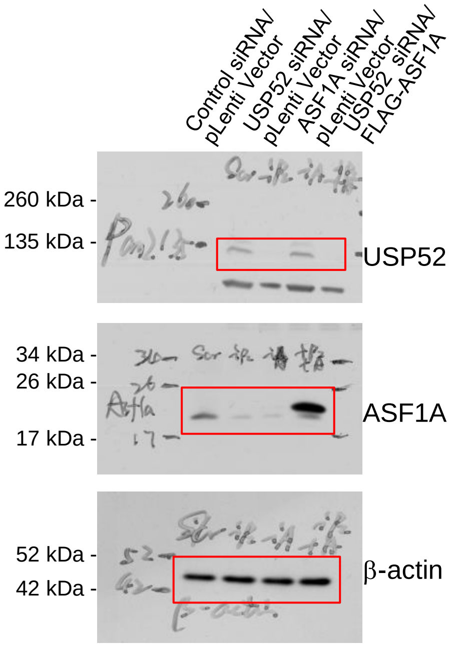

Uncropped blots related to Figure 6f

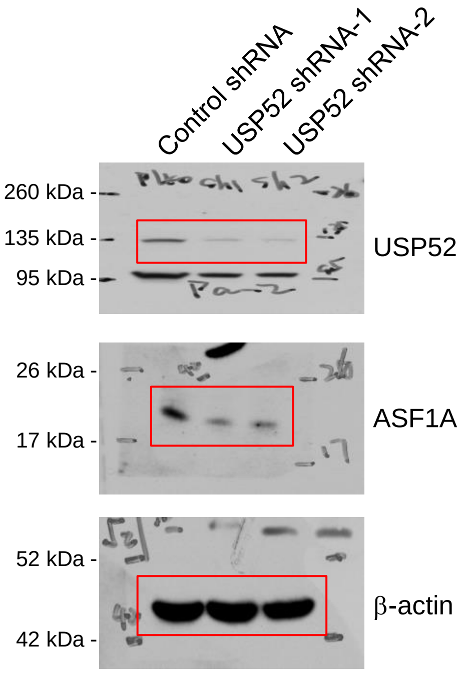

Uncropped blots related to Figure 7b

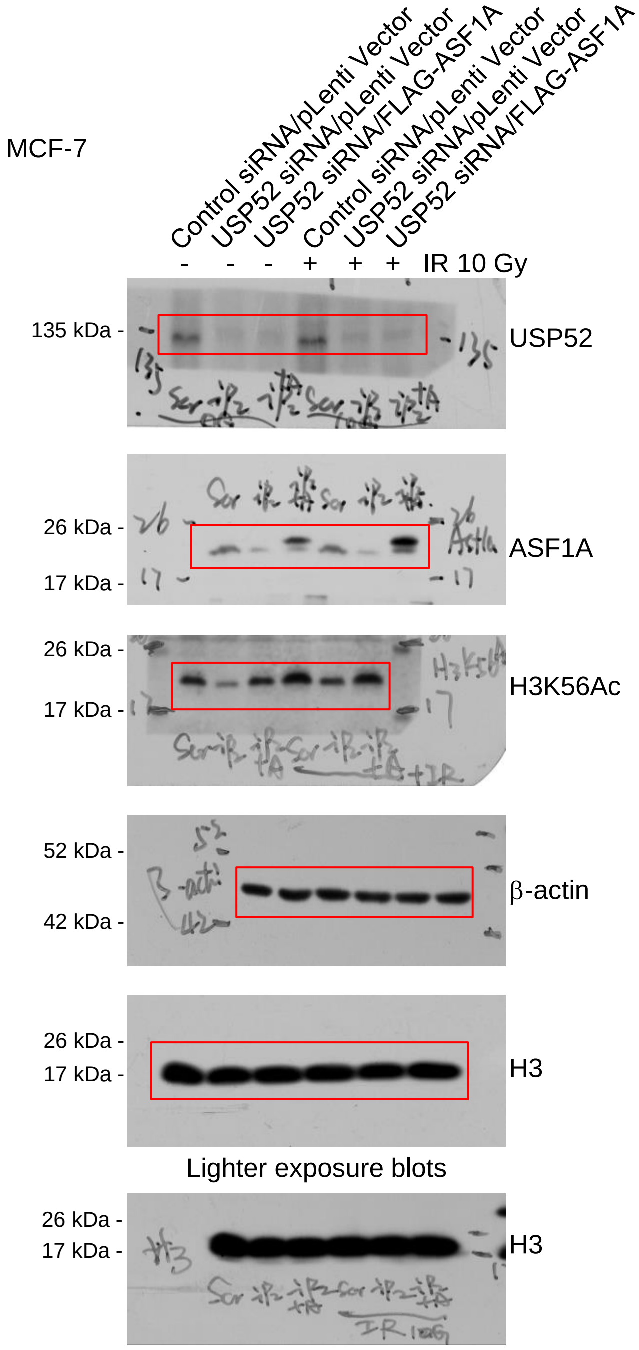

Longer exposure blots

Uncropped blots related to Figure 7c

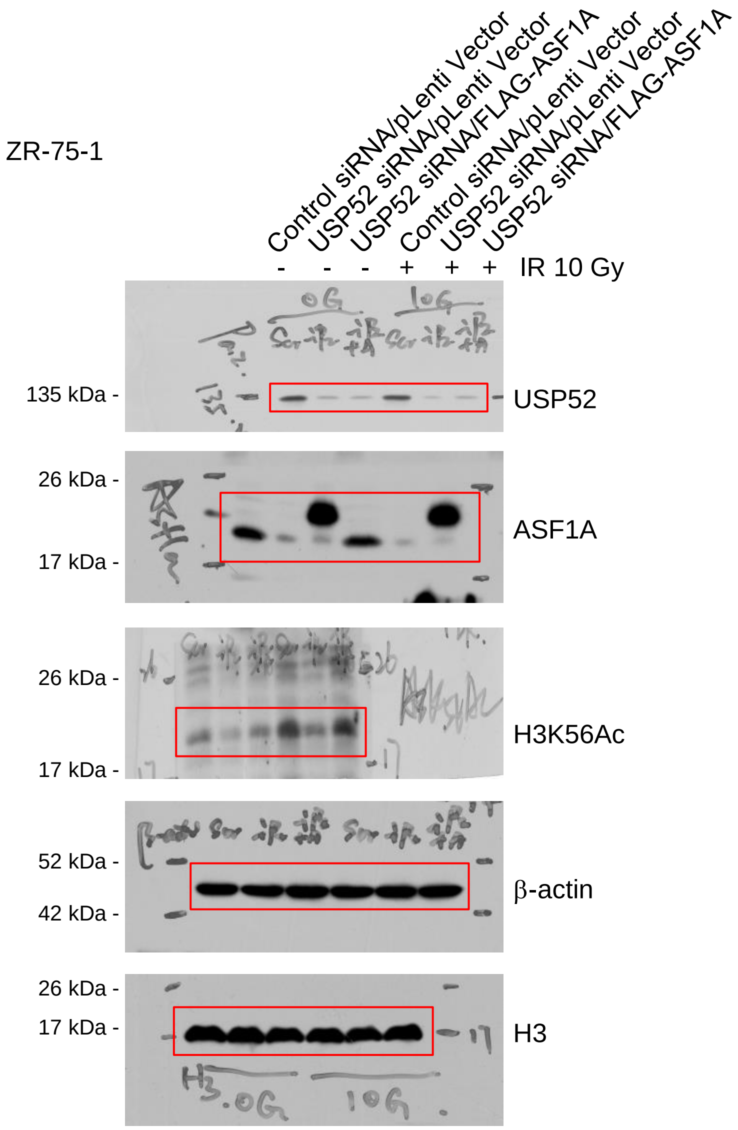

Supplementary Figure 8. Uncropped Scans for Figures 1-7. The relevant figures are indicated in the blot titles. In some blots, both lighter and longer exposed ones with markers are provided. The cropped areas within the red boxes are indicated.

Supplementary Table 1. siRNA Sequences

siRNAs	Sequences	
USP52-1	CUGUCUACCUGUUCCAUAUdTdT	AUAUGGAACAGGUAGACAGdTdT
USP52-2	GCAUGAUCCAGGUGCUCUAdTdT	UAGAGCACCUGGAUCAUGCdTdT
USP52-3	GAGUUACGCAGUGAUGGUAdTdT	UACCAUCACUGCGUAACUCdTdT
ASF1A-1	GGCAUAUGUUUGUAUUUCAdTdT	UGAAAUACAAACAUAUGCCdTdT
ASF1A-2	CAAUGUGAAGAAUUUGUUUdTdT	AAACAAAUUCUUCACAUUGdTdT
USP7-1	GACGUUUCGAAUAGAGGAAdTdT	UUCCUCUAUUCGAAACGUCdTdT
USP7-2	GGACUAUGACGUGUCUCUUdTdT	AAGAGACACGUCAUAGUCCdTdT

Supplementary Table 2. shRNA Sequences

shRNAs	Sequences
Control	CCGGGATATGGGCTGAATACAAACTCGAGTTTGTATTCAGCCCATATCTTTTTG
USP52-1	CCGGGCTTCCTTTCTCCATTCGCATCTCGAGATGCGAATGGAGAAAGGAAGCTTTTTG
USP52-2	CCGGGCTGGAGGACTTTGACTTCAACTCGAGTTGAAGTCAAAGTCCTCCAGCTTTTTG
USP52-3	CCGGCCTCTCCCTTTACTGTTCTATCTCGAGATAGAACAGTAAAGGGAGAGGTTTTTG (3'UTR)
Luciferase	TGATATGGGCTGAATACAAATTCAAGAGATTTGTATTCAGCCCATATCTTTTTTC
ASF1A-1	TGCGTAACTGTTGTGCTAATTTCAAGAGAATTAGCACAACAGTTACGCTTTTTTC
ASF1A-2	TGGCATATGTTTGTATTTCATTCAAGAGATGAAATACAAACATATGCCTTTTTTC

Note: Red color indicates the targeting sequence against the corresponding genes.

Supplementary Table 3. qRT-PCR Primers

Genes	Sequences
ACTB	F: CATGTACGTTGCTATCCAGGC
	R: CTCCTTAATGTCACGCACGAT
USP52	F: GTGGGTGTACCTGTTTCCGTC
	R: GCTCTGGATCTGCCGAATATCA
ASF1A	F: ATGCAGATGCAGTAGGCGTAA
	R: ACCCTGGGATTAGATGCCAAAA
