# Supplementary material for: USP52 acts as a deubiquitinase and promotes histone chaperone ASF1A stabilization
Source: Nat Commun. 2018 Mar 29;9:1285. doi: 10.1038/s41467-018-03588-z (PMC5876348; doi:10.1038/s41467-018-03588-z)
Supplement: Supplementary file 2 — Description of Additional Supplementary Files(PDF 172 kb) [file 41467_2018_3588_MOESM2_ESM.pdf]

Description of Additional Supplementary Files

File Name: Supplementary Data 1
Description: Mass Spectrometry Analysis of ASF1A-containing Protein Complex. Whole-cell extracts from MCF-7 cells with doxycycline (Dox)-inducible expression of stably integrated FLAG-ASF1A were purified with an anti-FLAG affinity column and analyzed by mass spectrometry. Information on Peptide fragments, peptide coverage and other parameters is shown.

File Name: Supplementary Data 2
Description: Mass spectrometry analysis of ASF1A ubiquitin conjugation sites. HeLa cells stably expressing FLAG-ASF1A were co-transfected with HA-Ub and USP52 siRNA. Cellular extracts were collected and sequentially purified with anti-FLAG affinity gel and HA affinity gel to enrich HA-Ub conjugated ASF1A. After trypsinization, the retrieved peptides were subjected to mass spectrometry analysis. Parameters of the identified ASF1A peptides with or without di-Glycine remnant are shown.
